# Bio-fabricated zinc oxide nanoparticles mediated by endophytic fungus *Aspergillus* sp. SA17 with antimicrobial and anticancer activities: *in vitro* supported by *in silico* studies

**DOI:** 10.3389/fmicb.2024.1366614

**Published:** 2024-05-13

**Authors:** Sally El Said Abo Halawa Abdelrahman, Seham El Hawary, Engy Mohsen, Mohamed A. El Raey, Heba Mohammed Refat M. Selim, Ahmed M. E. Hamdan, Mosad A. Ghareeb, Ahmed A. Hamed

**Affiliations:** ^1^Pharmacognosy Department, Faculty of Pharmacy, Cairo University, Giza, Egypt; ^2^Department of Phytochemistry and Plant Systematics, Pharmaceutical Division, National Research Centre, Cairo, Egypt; ^3^Department of Pharmaceutical Sciences, Faculty of Pharmacy, Almaarefa University, Riyadh, Saudi Arabia; ^4^Microbiology and Immunology Department, Faculty of Pharmacy (Girls), Al-Azhar University, Cairo, Egypt; ^5^Department of Pharmacy Practice, Faculty of Pharmacy, University of Tabuk, Tabuk, Saudi Arabia; ^6^Medicinal Chemistry Department, Theodor Bilharz Research Institute, Giza, Egypt; ^7^Microbial Chemistry Department, National Research Centre, Giza, Egypt

**Keywords:** *Aspergillus* sp. SA17, ZnONPs, antimicrobial, anticancer, UPLC-QTOF-MS/MS, docking, DNA gyrase, phosphoinositide 3-kinase gamma

## Abstract

**Introduction:**

In recent years, the world’s attention has been drawn to antimicrobial resistance (AMR) because to the frightening prospect of growing death rates. Nanomaterials are being investigated due to their potential in a wide range of technical and biological applications.

**Methods:**

The purpose of this study was to biosynthesis zinc oxide nanoparticles (ZnONPs) using *Aspergillus* sp. SA17 fungal extract, followed by characterization of the produced nanoparticles (NP) using electron microscopy (TEM and SEM), UV-analysis, X-ray diffraction (XRD), and Fourier-transform infrared spectroscopy (FT-IR).

**Results and Discussion:**

The HR-TEM revealed spherical nanoparticles with an average size of 7.2 nm, and XRD validated the crystalline nature and crystal structure features of the generated ZnONPs, while the zeta potential was 18.16 mV, indicating that the particles’ surfaces are positively charged. The FT-IR was also used to identify the biomolecules involved in the synthesis of ZnONPs. The antibacterial and anticancer properties of both the crude fungal extract and its nano-form against several microbial strains and cancer cell lines were also investigated. Inhibition zone diameters against pathogenic bacteria ranged from 3 to 13 mm, while IC_50_ values against cancer cell lines ranged from 17.65 to 84.55 M. Additionally, 33 compounds, including flavonoids, phenolic acids, coumarins, organic acids, anthraquinones, and lignans, were discovered through chemical profiling of the extract using UPLC-QTOF-MS/MS. Some molecules, such pomiferin and glabrol, may be useful for antibacterial purposes, according to *in silico* study, while daidzein 4’-sulfate showed promise as an anti-cancer metabolite.

## Introduction

Nanoparticles have gained significant attention due to their unique physiochemical properties which permit them to conjugate other groups due to their charged surface (Chavan et al., 2020; Randive et al., 2020, 2021, 2023). Among these nanoparticles, Zinc nanoparticles (ZnONPs) have gained reasonable interest in recent years due to their unique properties such as their small size and large surface which make them ideal candidates for use in numerous fields like biomedicine, electronics, and agriculture (Gomaa, 2022). Biosynthesis of ZnONPs from endophytic fungi has emerged as a promising alternative way to produce eco-friendly and cost-effective nanometals compared to conventional physical and chemical methods (Yusof et al., 2019).

Several studies have reported the biosynthesis of ZnONPs using endophytic fungi such as *Fusarium oxysporum*, *Aspergillus fumigatus*, and *Trichoderma viride*. The biosynthesis of ZnONPs using endophytic fungi is achieved by reducing the metal ions to their corresponding nanoparticles, which are stabilized by various biomolecules such as proteins, polysaccharides, and enzymes produced by the fungi (Bachheti et al., 2021). ZnONPs have been extensively studied for their potential applications as antimicrobial agents with a broad spectrum toward a large number of pathogens including bacteria, fungi, and viruses (Abdelaziz et al., 2021). ZnONPs have been shown to have antibacterial properties against a variety of harmful microorganisms, including *Pseudomonas aeruginosa*, *Staphylococcus aureus*, and *Escherichia coli.* Furthermore, it has been claimed that the death of cancer cells could possibly be achieved by using ZnONPs through variable pathways, *viz.* autophagy, necrosis, and apoptosis. ZnONPs have been shown to exhibit anticancer properties against various cancer cell types, including those found in the prostate, lung, and breast tissues (El-Bendary et al., 2021; Abdel-Nasser et al., 2022).

However, no published data currently exist regarding the antimicrobial and anticancer properties of the characterized ZnO nanoparticles with *Aspergillus* sp. SA17 crude extract. This lack of information has motivated us to explore the phytochemical identity of the crude extract, considering their previously reported data and using molecular docking. Molecular docking is an important computational technique to predict the best interaction between ligand and its functional site.

The objectives of this study were the biosynthesis and characterization of ZnO nanoparticles using *Aspergillus* sp. SA17 crude extract. Moreover, the chemical constituents of the crude extract were identified using UPLC-QTOF-MS/MS. The antimicrobial and anticancer activities of the crude extract and its nano-form were evaluated to assess the underlying mechanisms by which crude extract inhibited cell proliferation. Also, a molecular docking study was performed.

## Materials and methods

### Fungal isolation

The separation of endophytic fungi from marine seagrass was performed using the surface sterilization method. The marine seagrass was collected from Hurghada, Egypt, and washed thoroughly with running seawater to remove debris and sand. The samples were then cut into small pieces and surface sterilized via immersion in 70% ethanol for 30 s, followed by washing three times with sterile seawater, then immersion in 5% sodium hypochlorite for 30 s, and then washing three times with sterile seawater. After surface sterilization, the seagrass samples were cut and placed on a potato dextrose agar (PDA) medium supplemented with antibiotics (ampicillin 100 mg/L and streptomycin 50 mg/L) to inhibit bacterial contamination. The plates were incubated at 25°C until fungal colonies appeared (Li and Wang, 2009).

### Genetic identification of fungal strain

The fungus strains were isolated and cultivated for 5 days at 25°C in potato dextrose broth media. The suspension was then centrifuged at 10,000 rpm for 10 min at room temperature. DNA extraction was carried out using DNeasy Blood & Tissue Kits manufactured by Qiagen, a leading biotechnology company headquartered in Hilden, Germany. Extraction was performed according to the manufacturer’s instructions. The kit is designed to efficiently purify genomic DNA from various sample sources. Amplification was carried out using two primers, ITS2, GCTGCGTTCTTCATCGATGC, and ITS3, GCATCGATGAAGAACGCAGC (White et al., 1990) and the PCR condition was as follows: Denaturation for 5 min at 94°C, followed by 35 cycles of 30 s at 94°C, then 30 s at 55°C, 90 s at 72°C, and a final 5 min extension step at 72°C. The purified PCR product was sequenced using two primers: ITS1, TCCGTAGGTGAAC-CTGCGG, and ITS4, TCCTCCGCTTATTGATATGC (Op De Beeck et al., 2014; Hamed et al., 2020; Elawady et al., 2023; Khazaal et al., 2023).

### Fungal filtrate preparation

The fungus was cultivated in potato dextrose (PD) broth. The PD broth was created by dissolving 24 g of potato dextrose broth (PDB) in 1 L of distilled water. The medium was then poured into sterile flasks. The flasks were sealed with cotton plugs and sterilized by autoclaving at 121°C for 15 min. The endophytic fungi were inoculated into the PD broth by transferring a small piece of fungal mycelium from the PDA plate into the broth using a sterile loop. The flasks were then incubated at 29°C for 7 days with constant shaking at 150 rpm. After 7 days of cultivation, the fungal mycelia were removed from the broth by filtration through filter paper. The filtrate was then gathered and centrifuged at 10,000 rpm for 10 min to eliminate any residual fungal cells or debris. The resulting supernatant was used for further analysis.

### The biosynthesis of zinc oxide nanoparticles

Specifically, 1 g of the hydroalcoholic extract was blended with 5 g of a zinc acetate solution, which was dissolvable in 500 mL of bi-distilled water (Attia et al., 2021; Alrabayah et al., 2022). The resulting mixture was heated under stirring for 20 min at 80°C. After that NH_4_OH drops were added till the formation of yellowish white sediment. The blend was then allowed to sit for 30 min to finalize the interaction. The resulting sediment was centrifuged at 4,000 rpm, cleaned twofold with bi-distilled water, and then cleaned with EtOH to yield a pale yellowish powder of ZnONPs (Ghareeb et al., 2019a; Eskander et al., 2020; Elkhouly et al., 2021a; Hameed et al., 2023).

### Characterization of ZnONPs

#### UV-spectroscopy

The ultraviolet–visible spectral examination was performed using a UV spectrophotometer (Shimadzu Corporation, Japan) to track the transformation of the zinc ion to zinc oxide nanoparticles. The UV spectrum was recorded between 200 and 800 nm.

#### Fourier-transform infrared spectroscopy

To characterize the functional groups that participated in ZnONPs formation, Fourier transform infrared (FT-IR) analysis was conducted utilizing a FT-IR 6100 spectrometer (Jasco, Japan) operating at a temperature of 25°C, in the domain from 4,000 to 400 cm^−1^. FT-IR spectroscopy is a potent analytical technique used in nanoparticle characterization because of its ability to identify individual chemical bonds and functional groups in the sample. FT-IR spectroscopy sheds light on molecular structure, composition, and interactions by evaluating the sample’s absorption or emission of infrared radiation. During the analysis, the FT-IR spectrometer fired infrared light at the ZnONPs sample, and the resulting spectrum was recorded. Peaks and patterns in the spectrum corresponded to typical vibrations of chemical bonds within the nanoparticles, such as stretching and bending modes of functional groups such as -OH (hydroxyl), C=O (carbonyl), C-H (alkyl), and others.

#### Zeta potential measurements

The constancy and charges of the synthesized nanoparticles were assessed using a zeta sizer nano-z’s laser diffractometer (Malvern, United Kingdom). The zeta sizer nano-z’s is equipped with dynamic light scattering (DLS) technology and operates at a temperature of 25°C. Zeta potential measures the electrostatic repulsion forces inside nanostructures and is an important metric for evaluating colloidal stability.

#### X-ray diffraction

X-ray diffraction (XRD) was carried out using a Bruker D8 Advance Diffractometer (Bruker AXS, Germany) with Cu Ka radiation (k = 1.54) to obtain the XRD pattern of ZnONPs over a 2-theta range of 10–90. XRD detecting and verifying the crystal structure of nanoscale materials, XRD is helpful. The arrangement of atoms in the crystalline lattice can be inferred from the diffraction patterns produced by X-ray diffraction (XRD), which provides important details on the characteristics of the material. The XRD experiment measured a 2-theta range of 10° to 90°. This broad range enabled a complete assessment of the diffraction configurations generated by the ZnONPs, allowing the study of the crystallographic characteristics. X-ray diffraction is operated based on Bragg’s law, with X-rays impacted on a crystalline material diffracted at certain angles given by the spacing of atomic planes inside the crystal lattice. The resulting diffraction pattern contains information on the atoms’ arrangement in the crystal structure, such as lattice parameters, crystal orientation, and phase purity. Using the XRD pattern acquired from the ZnONPs sample, researchers were able to determine the crystal phases present, their relative abundance, and the degree of crystallinity.

#### Electron microscopy

Finally, the particle dimension and shape of the zinc oxide nanoparticles were examined utilizing transmission electron microscopy (TEM) with a JEOL-JEM-1011 instrument (Japan). Drops of the suspended nanoparticle solution were located on a carbon-coated copper grid, and the solvent was permitted to outlet gradually before the TEM picture was recorded. Additionally, Furthermore, the morphology of the biosynthesized zinc oxide nanoparticles was further studied using scanning electron microscopy (SEM; Quanta FEG-250, Netherlands).

### Biological activity

#### Antimicrobial activity

The antimicrobial activity of the crude extract and ZnONPs was evaluated vs. some pathogenic microbial strains *Staphylococcus aureus*, *Bacillus subtilis*, *Escherichia coli*, *Pseudomonas aeruginosa*, *Candida albicans,* and *Aspergillus flavus* according to the reported procedures (Madkour et al., 2017; Khalaf et al., 2019). The tested samples were dissolved in methanol and a solution of the concentration 1 mg /ml was prepared separately. Paper discs of Whatman filter paper were prepared in standard size (5 cm) and were cut and sterilized in an autoclave. The paper discs soaked in the desired concentration of the complex solution were placed aseptically in the petri dishes containing nutrient agar media (agar 20 g + beef extract 3 g + peptone 5 g) seeded with the tested microbial strains. The petri dishes were incubated at 36°C and the inhibition zones were recorded after 24 h of incubation. Each treatment was replicated three times. Ampicillin and Clotrimazole were utilized as standard antibiotics. The activity of the standard antibiotics was also recorded using the above-mentioned procedures at the same concentration and solvents. The % efficacy index was estimated using the formula:


$$\%\mathrm{Activity}\;\mathrm{Index}=\frac{\mathrm{Zone}\;\mathrm{of}\;\mathrm{inhibition}\;by\;\mathrm{test}\;\mathrm{sample}\;\left(\mathrm{diametre}\right)}{\mathrm{Zone}\;\mathrm{of}\;\mathrm{inhibition}\;by\;\mathrm{standard}\;\left(\mathrm{diametre}\right)}$$

#### Cytotoxicity (MTT assay)

The cytotoxic activity of the tested samples was performed using MTT assay according to the reported procedures (Saad et al., 2017; Ghareeb et al., 2020) using five cell lines namely; human lung fibroblast (WI38), colorectal carcinoma colon cancer (HCT-116), mammary gland breast cancer (MCF-7) and hepatocellular carcinoma (HEPG-2). Doxorubicin was utilized as a standard anticancer drug. The cell lines were obtained from ATCC via Holding company for biological products and vaccines (VACSERA), Cairo, Egypt. This assay is based on the conversion of the yellow tetrazolium bromide (MTT) to a purple formazan derivative by mitochondrial succinate dehydrogenase in viable cells. Cell lines were cultured in RPMI-1640 medium with 10% fetal bovine serum. Antibiotics added were 100 units/mL penicillin and 100 μg/mL streptomycin at 37°C in a 5% CO_2_ incubator. The cell lines were seeded in a 96-well plate at a density of 1.0 × 10^4^ cells/well. at 37°C for 48 h under 5% CO_2_. After incubation, the cells were treated with different concentrations of samples and incubated for 24 h. After 24 h of drug treatment, 20 μL of MTT solution at 5 mg/mL was added and incubated for 4 h. Dimethyl sulfoxide (DMSO) in a volume of 100 μL is added into each well to dissolve the purple formazan formed. The colorimetric assay is measured and recorded at the absorbance of 570 nm using a plate reader (EXL 800, United States). The relative cell viability in percentage was calculated as (A570 of treated samples/A570 of untreated sample) × 100.

#### UPLC–MS/MS analysis

UPLC analysis was performed via using Shimadzu ExionLC system with the following conditions “Mobile phase A: 0.1% formic acid in water and mobile phase B: acetonitrile; Column: GL-Science (100*2.1) mm, 3 μm; Flow rate: 0.35 mL \min; Column oven: 50°C; Time gradient/Mobile phase B %: 0.0/5.0, 5.0/5.0, 25.0/95.0, 30.0/95.0, 32.0/5.0, and 40.0/5.0. The mass instrument specifications are “Instrument Model: X500 QTOF, Source name: TurboIonSpray, *Curtain gas (psi):* 30, Ion source gas 1 (psi): 50, Ion source gas 2 (psi): 50, Temperature (°C): 500, Scan type: Full scan -SWATH-Screening, Ion spray voltage (V): Negative, −4,000 V, and CAD gas: 7 (Mahmoud et al., 2023).

### *In silico* study

#### Virtual target identification

The putative target characterization was achieved via Pharmacophore-based Virtual screening using PharmMapper (Wang et al., 2017; El-Sayad et al., 2023). This platform assigns a score to each molecule in the PDB that best fits a pharmacophore model that has been extracted and stored as a library of ligand dataset in mol2 format. After that, when a new molecule is submitted, its fit score is calculated for each pharmacophore, and then each fit score for that pharmacophore is compared to the fit score matrix to determine where it falls on the scale of all the pharmacophore scores. In comparison to chance pharmacophore matching, the pure fit score that results from this procedure carries considerably more weight and assurance. The query structure was submitted to the platform in the PDB format, and the retrieved results were exported as Excel sheet arranging the resulted protein targets according their fit scores.

#### Docking study

The crystal structures of *E. coli* GyrB (PDB ID: 6kzv), and both the human PI3K-γ and c-Src (PDB ID: 2v4l and 3en7, respectively) were used for the docking study using AutoDock Vina (Huey et al., 2012). The co-crystallized ligand in each structure was used to determine the binding site and the docking grid-box in each protein structure, respectively. The co-ordinates of the grid-box were set to be: x = −7.86, y = 16.12, z = 2.49; and x = 45.07, y = 13.12, z = 31.49; and x = −5.09, y = 6.34, z = −6.66, respectively. The ligand to binding site shape matching root means square (RMSD) threshold was set to 2.0 Å. The interaction energies were determined using the Charmm force field (v.1.02) with 10.0 Å as a non-bonded cutoff distance and distance-dependent dielectric. Then, 5.0 Å was set as an energy grid extending from the binding site (Huey et al., 2012). The tested compound retinol was energy minimized inside the selected binding pocket. The editing and visualization of the generated binding poses were performed using Pymol software (Yuan et al., 2017).

#### Molecular dynamics simulation

NAMD 3.0.0. software was used for performing MDS (Yuan et al., 2017; Ribeiro et al., 2018; Soltane et al., 2023). This software applies the Charmm-36 force field. Protein systems were built using the QwikMD toolkit of the VMD software (Humphrey et al., 1996; Ribeiro et al., 2018), where the protein structures were checked for any missing hydrogens, the protonation states of the amino acid residues were set (pH = 7.4), and the co-crystalized water molecules were removed. Thereafter, the whole structures were embedded in an orthorhombic box of TIP3P water together with 0.15 M Na^+^ and Cl^−^ ions in 20 Å solvent buffer. Afterward, the prepared systems were energy-minimized and equilibrated for 5 ns. The parameters and topologies of the ligands were calculated by using the VMD plugin Force Field Toolkit (ffTK). Afterward, the generated parameters and topology files were loaded to VMD to readily read the protein–ligand complexes without errors and then conduct the simulation steps.

#### Binding free energy calculations

Molecular Mechanics Poisson-Boltzmann Surface Area (MM-PBSA) embedded in the MMPBSA.py module of AMBER18 was utilized to calculate the binding free energy of the docked complex (Humphrey et al., 1996; Ghareeb et al., 2024). 100 frames were processed from the trajectories in total, and the system’s net energy was estimated using the following equation:


$$\Delta G_{\mathrm{Binding}}=\Delta G_{\mathrm{Complex}}-\Delta G_{\mathrm{Receptor}}-\Delta G_{\mathrm{Inhibitor}}$$

Each of the aforementioned terms requires the calculation of multiple energy components, including van der Waals energy, electrostatic energy, internal energy from molecular mechanics, and polar contribution to solvation energy.

### Statistical analysis

Results were analyzed statistically using the computerized program SPSS software, version “20” for windows. The one-way analysis of variance “ANOVA” test was done followed by Duncan test. Data were represented as mean ± SE. Values that are less than 0.05 were considered significant, otherwise were non-significant.

## Results

### Isolation of the fungal isolate from different marine samples

**Figure 1:**
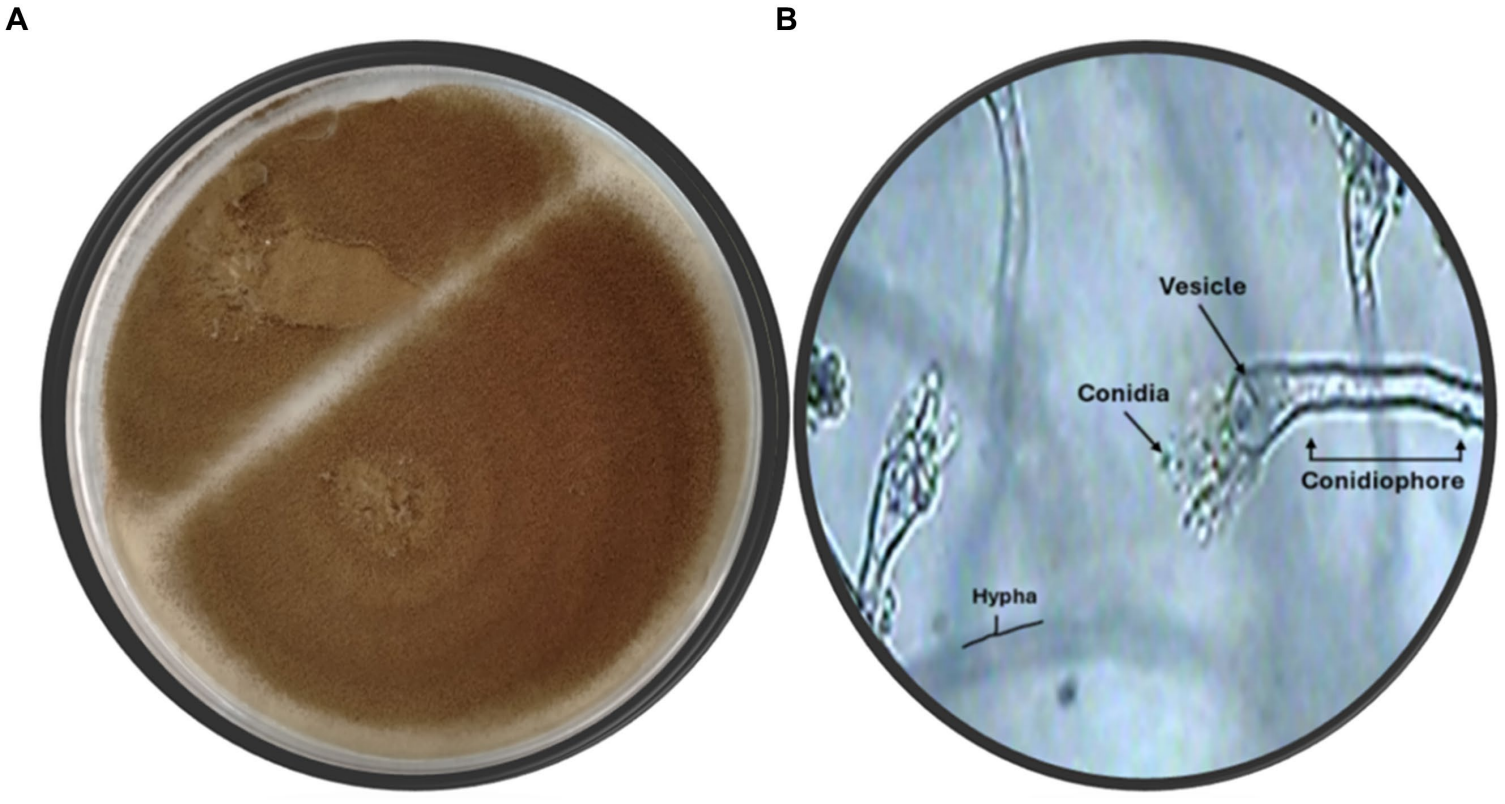
**(A)** Fungus color on agar plate **(B)** Microscopic identification of isolate SA17 showing the conidia, vesicle, hypha, and conidiophore of the fungus magnification x500.

The fungal strain SA17 was isolated and purified from the collected seagrass samples. The fungal colony was selected based on its morphological features. The obtained strain was deposited at 4°C at the Microbial Chemistry Department, National Research Centre, Egypt. Preliminary identification of the isolated fungal isolate SA17 was carried out based on its morphological characteristics. The isolate showed fast-growing colonies with a cottony green appearance. It produces conidiophores that bear numerous conidia (Figure 1).

**Figure 2:**
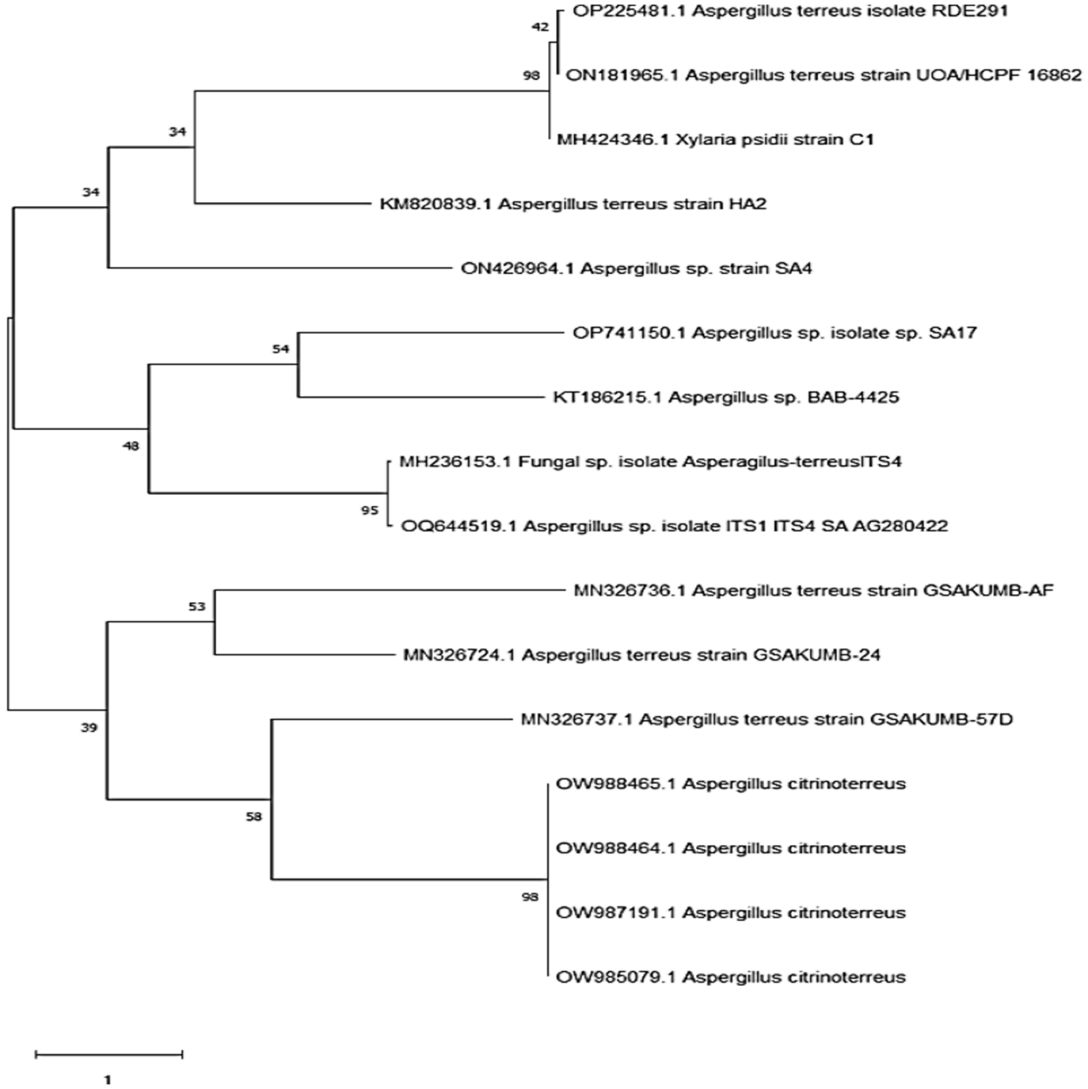
Phylogenetic tree of the *Aspergillus* sp. SA17.

The identification of the fungal strain was performed using sequencing of the 18S rRNA gene depending on the initial assessment of the isolated fungi. Subsequently, the DNA of the fungal sample was extracted, amplified, and identified by matching it with other known deposited genes in the GeneBank database using the BLAST approach to define the symmetry record and statistical value of the matches (https://blast.ncbi.nlm.nih.gov/Blast.cgi, accessed on 1 February 2023). The findings disclosed that the 18S rRNA gene arrangements of the isolate were identical, with the *Aspergillus* sp. having 100% symmetry. The evolution registry (Figure 2) was estimated via the Maximum Likelihood method and the Tamura–Nei model (Humphrey et al., 1996). The proportion of trees in which the correlated taxa are gathered is shown after the branches. The Tamura–Nei model was utilized to produce a matrix of pairwise distances, and the topology with the maximum log-likelihood value was selected as the first tree for the heuristic inspection. MEGA X was utilized to perform the evolutionary exploration (Miller III et al., 2012).

### Preparation of fungal crude filtrate and bio-synthesis of zinc oxide nanoparticles

After the fungal culture had grown to the desired phase, the filtrate was obtained by filtering the culture through a sterilized filter paper. The obtained filtrate was mixed with a solution containing zinc ions (zinc acetate). The mixture is then incubated at a specific temperature for a certain period. During this time, the enzymes and biomolecules present in the fungal filtrate act as reducing agents, converting the zinc ions into zinc oxide nanoparticles in solution after heating at 100°C. The dimension and shape of the nanoparticles can be managed by altering the concentration of the precursor solution, the incubation time, and the temperature.

### Characterization

#### UV-analysis

**Figure 3:**
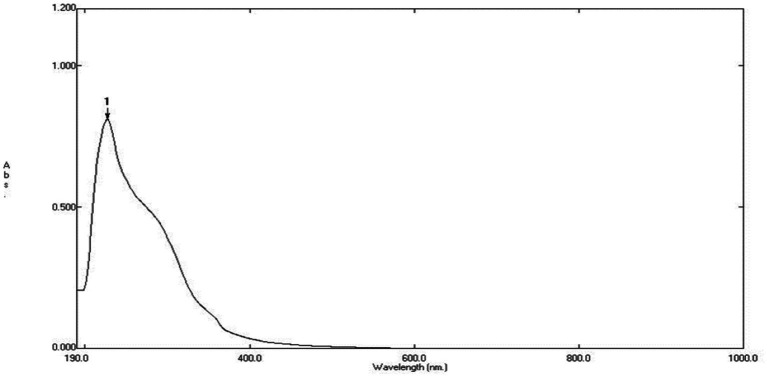
UV spectrum of biosynthesized ZnO nanoparticles.

The UV–visible spectroscopy was utilized to monitor the green synthesis of ZnONPs using an aqueous fungal extract. Upon addition of the fungal extract to the filtered zinc acetate solution, the color of the solution changed from brownish red to pale yellow, indicating the successful reduction of Zn(CH_3_COO)_2_ to zinc oxide nanoparticles. The synthesized ZnONPs were then characterized by UV–Vis spectroscopy, which revealed a wide absorption band at 280 and 340 nm as shown in Figure 3, confirming the formation of ZnONPs.

The produced ZnONPs were examined using UV–visible spectroscopy, which is the accepted technique for confirming nanoparticle creation and evaluating their optical characteristics. This method works especially well for “green synthesis,” wherein extracts derived from biological processes serve as reducing agents in metal nanoparticles biosynthesis. The study’s conclusions-which are supported by UV–Vis spectroscopy-highlight the usefulness of the fungal extract in the environmentally friendly synthesis of ZnONPs and call attention to its possible uses in the production of sustainable nanomaterials (Harris et al., 2016).

#### Fourier transforms infrared spectroscopy analysis

**Figure 4:**
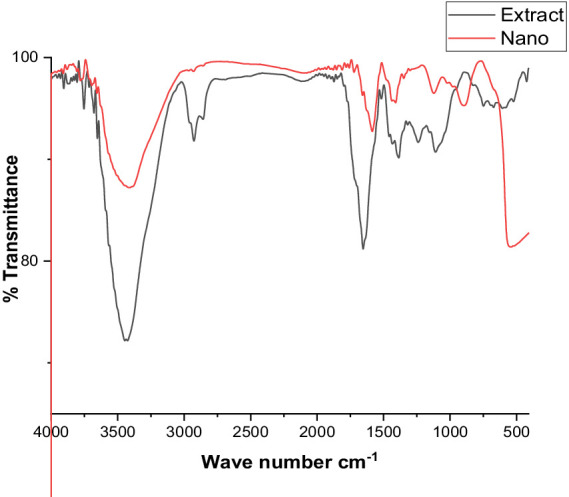
FTIR spectra for the crude extract, and ZnO nanoparticles.

The FT-IR analysis of the extract and the produced ZnO nanoparticles is shown in Figure 4. Here, FT-IR analysis has been used to identify the functional groups in charge of ZnONPs production and stability. The FT-IR spectra of the green ZnONPs showed that the various functional groups, CH, C=O, C=C, C-O, C-N, and C-C- in the synthesized ZnONPs were connected to peaks at 3500, 3000, 2,800, 1,700, 1,500, 1335.25, 1218.83, and 1030.65 cm^−1^. The stretching vibration of the hydroxyl group (O-H) is found at 3320, whereas the stretching vibration of the alkane (C-H) is found at peaks at 2919.92 and 2850.58 cm^−1^. Similarly, a stretch vibration of carbonyl (C=O) was detected at 1,700 cm^−1^. At 1730.72 cm^−1^, the aromatic (C=C) stretch bands were observed. Stretching of the C-N was mentioned in the band at 1,200 cm − 1, while the C-O was detected at 1,305. Furthermore, the C-C is located at 1030.65 cm^−1^. The presence of flavonoids and phenolic acids in the tested extract can be fundamentally linked to the occurrence of absorption bands related to C=C, C=O, C-O, C-N, OH, and CH (Wu et al., 2022). These functional groups contribute to the stability of the prepared ZnONPs as well as the reduction of Zn metal ions.

#### Zeta potential of synthesized ZnONPs

**Figure 5:**
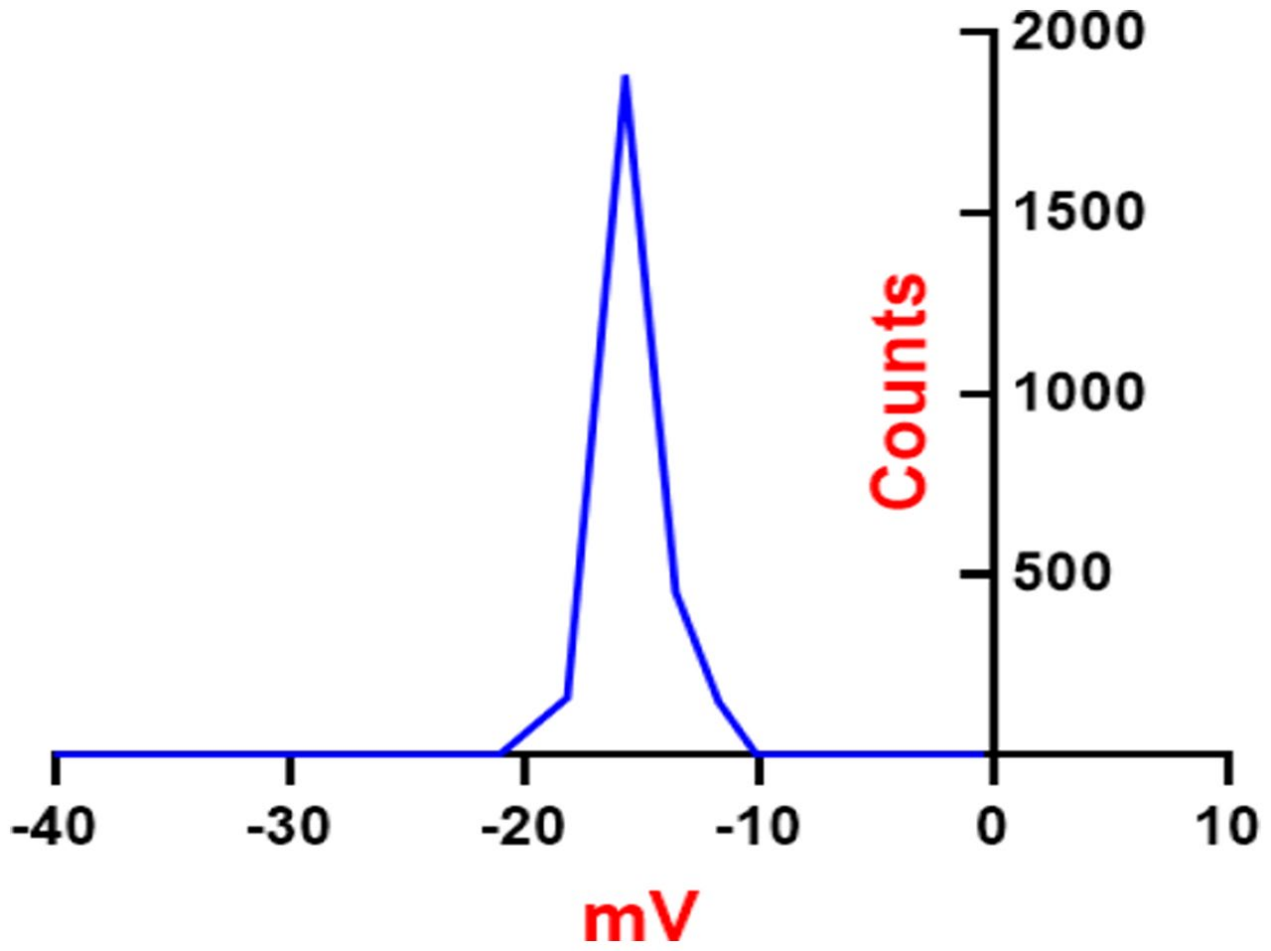
Zeta potential analysis of synthesized SA-17 ZnONPs.

ZnONPs colloidal solutions displayed constancy in the neutral aqueous system (di-distilled water) since it possesses a zeta potential value of −18.16 mV as presented in Figure 5. ZnONPs colloidal stability in a neutral media has important ramifications for possible uses. Stable colloidal dispersions are crucial for drug delivery in the biomedical domains because they inhibit aggregation and preserve the intended particle size distribution, which influences the kinetics of drug release and the effectiveness of targeting (Wilhelm et al., 2016). Furthermore, in environmental applications, the dispersion and interaction of nanoparticles with contaminants, which facilitates their removal and remediation from aqueous systems, are greatly aided by their colloidal stability. It is important to remember that there are additional variables that might affect colloidal stability than the zeta potential, including particle size, surface charge density, and the presence of stabilizing chemicals (Li et al., 2013).

#### X-ray diffraction pattern examination

**Figure 6:**
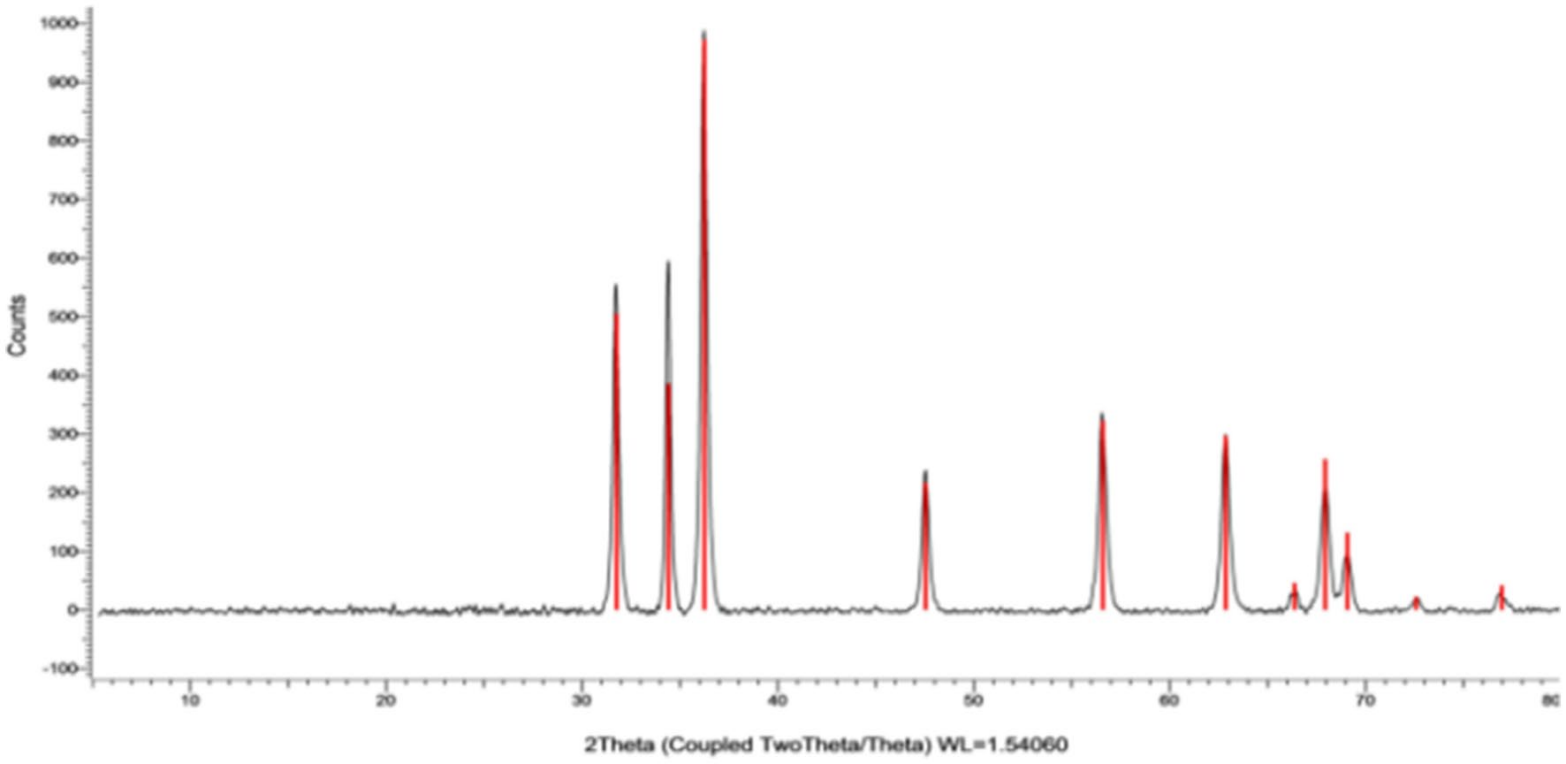
XRD spectrum of synthesized SA-17 ZnONPs.

The X-ray diffraction (XRD) paradigm was utilized to confirm the crystalline nature of ZnONPs. The XRD pattern displayed distinct peaks at (2θ) angles of 31.77, 34.43, 36.26, 47.55, 56.60, 62.88, 66.39, 67.96, and 69.10°, complemented by indicators (100), (002), (101), (102), (210), and (103), sequentially, as noted in Figure 6. The XRD spectra of ZnONPs revealed that the generated nano-ZnO had a spherical form and matched the profile mentioned by Schreyer et al. (2014).

#### Transmission and scanning electron microscopy studies

**Figure 7:**
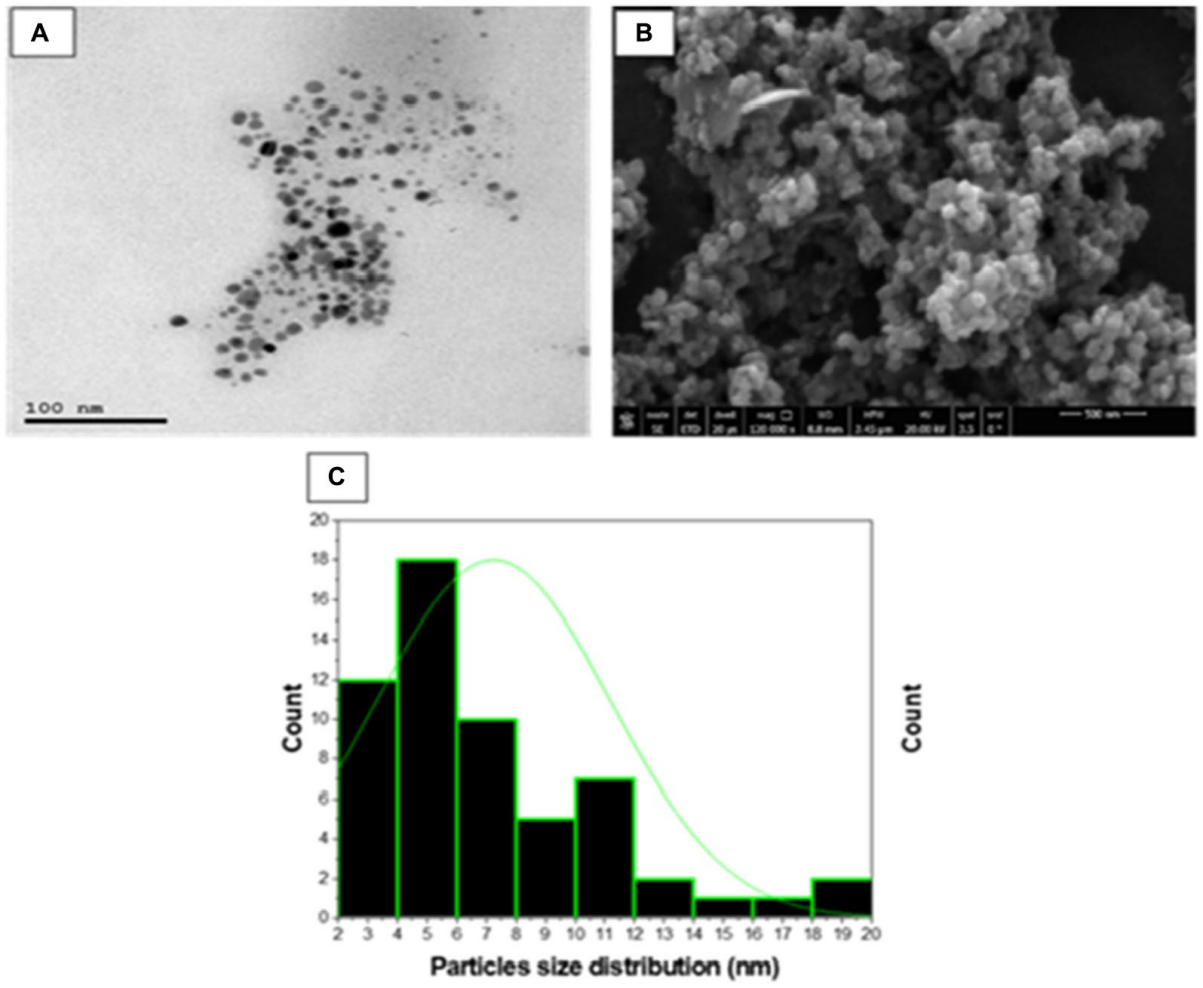
**(A)** TEM micrograph, **(B)** SEM micrograph and **(C)** Particle size distribution of synthesized SA-17 ZnONPs.

High resolution-TEM was done to investigate the nature of SA-17 ZnONPs particle size distribution and its crystallinity. As shown in Figure 7, the particles consist of a spherical crystal of particle size extending from 3 to 20 nm with an average of 7.2 nm. These spherical crystals contain small particles from 2 to 30 nm with average size of 28.9 nm. The surface morphology of the ZnONPs was explored using a captured SEM image, which showed that the ZnONPs were agglomerated spherical shapes with a diameter ranging from 13 to 55 nm with an average size of 30 nm (Figure 7B). The size detected by SEM was slightly larger than those by TEM as result of surface particles were agglomerated to each other.

#### Antimicrobial activity

**Figure 8:**
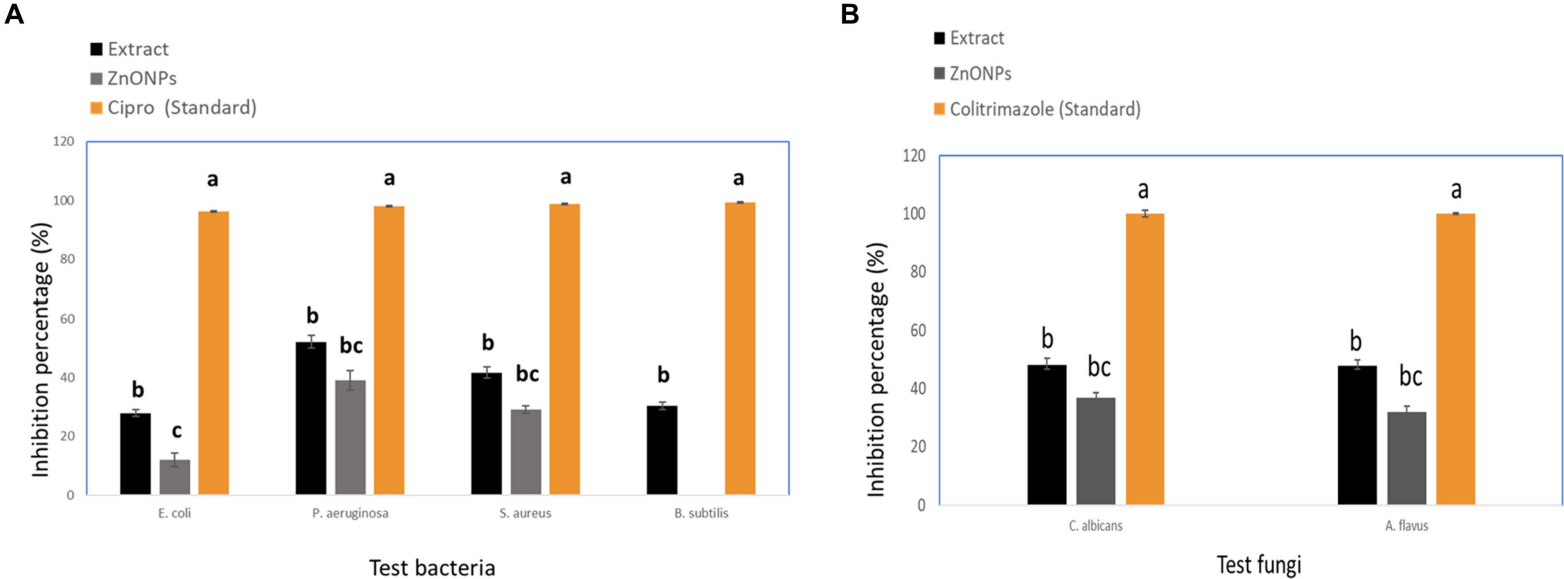
Inhibition percentage against pathogenic microbial strains **(A)** Tested bacteria, and **(B)** Tested fungi. While different lowercase letters meant that the antimicrobial effect was significantly different (*p* < 0.05).

The crude extract and biosynthesized ZnONPs showed a pronounced antimicrobial effect toward *E. coli*, *P. aeruginosa*, *S. aureus*, *B. subtilis*, *C. albicans*, and *A. flavus*, respectively. With inhibition percentages ranging from 96.25% to 99.26%, the data showed that Cipro had the highest antibacterial efficiency against all tested pathogens. By contrast, although to a lesser degree, the extract and ZnONPs likewise showed noteworthy antibacterial activity. ZnONPs showed inhibitory percentages ranging from 12.0% to 39.1%, and the extract showed percentages ranging from 28.0% to 52.2%. Significant variations in the antibacterial activity of the tested medicines against every bacterium were found by statistical analysis (*p* < 0.05). By contrast, the extract and ZnONPs both showed strong antifungal activity. ZnONPs displayed inhibitory percentages ranging from 37.0% to 32.0%, whereas the extract showed percentages ranging from 48.1% to 48.0%. The antifungal activity of the tested medicines against each microbe varied significantly, according to statistical analysis (*p* < 0.05). The following letters indicate statistical significance for *Aspergillus flavus* and *Candida albicans*: Colitrimazole (a) >Extract (b) >ZnONPs (c). The obtained results were compared with standard antibiotics, including Ciprofloxacin and Colitrimazole. *Aspergillus* species are known for producing unique and diverse secondary metabolites with potent biological activities (Zhang et al., 2018; Abdel-Razek et al., 2020). Figure 8 displayed the inhibition ratio against all tested microbes.

#### Anticancer activity

**Figure 9:**
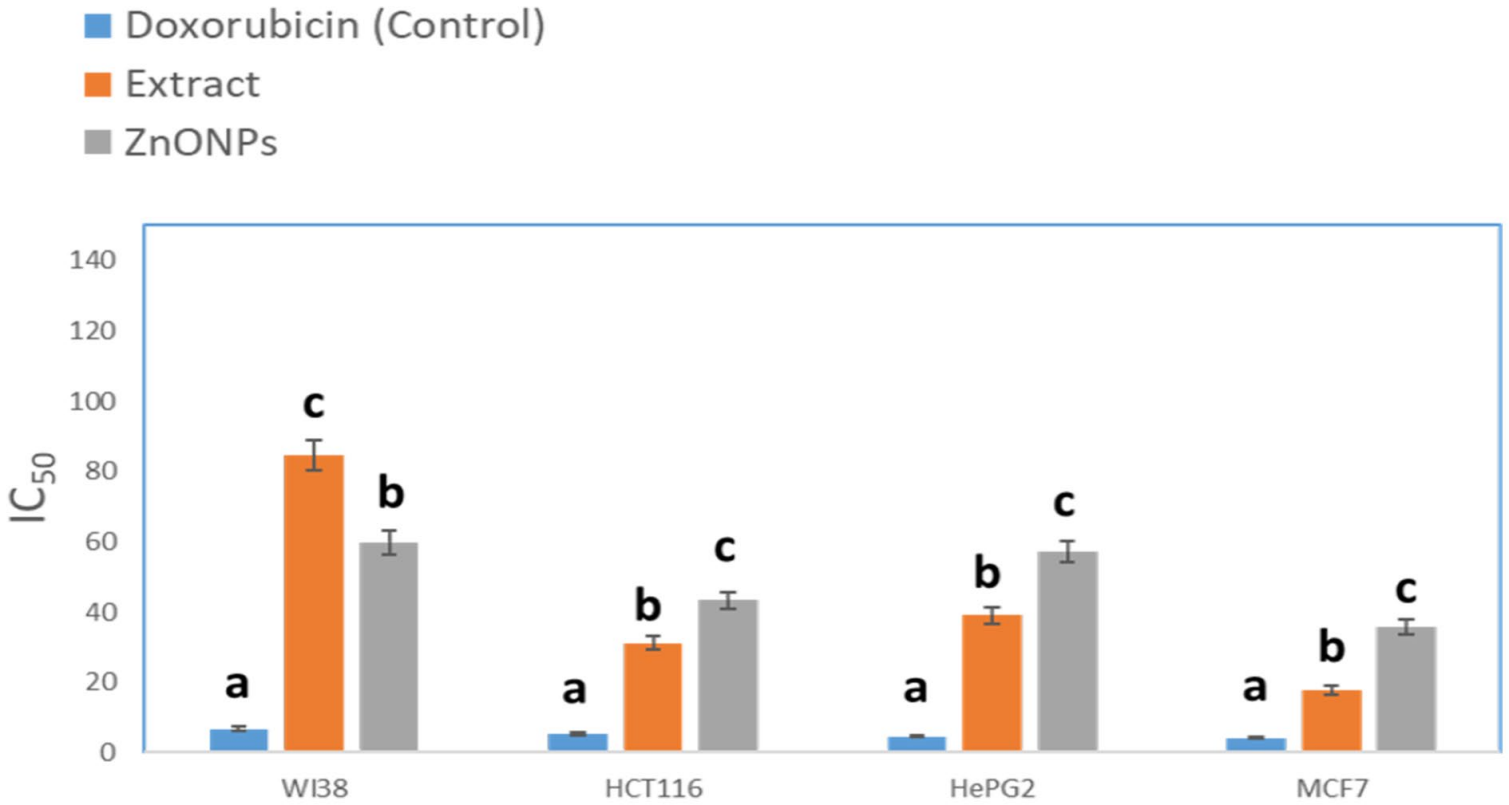
Anticancer activity of the tested extracts against human cell lines. While different lowercase letters meant that the anticancer effect was significantly different (*p* < 0.05).

The crude extract demonstrated anticancer activity with IC_50_ values of (84.55 μM), (31.13 μM), (39.06 mm), and (17.65 μM), against WI38, HCT116, HePG2, and MCF7, respectively. On the other hand, the biosynthesized ZnONPs displayed anticancer activity with IC_50_ values of (59.74 μM), (43.21 μM), (57.03 mm), and (35.66 μM), against WI38, HCT116, HePG2, and MCF7, respectively. The results were compared with Doxorubicin (Figure 9). The cytotoxicity of the tested drugs against each cell line varied significantly, according to statistical analysis (p < 0.05). The letters that indicate statistical significance for the cell lines WI38, HCT116, HePG2, and MCF7 are as follows: ZnONPs (c) > Extract (b) > Doxorubicin (a). *Aspergillus* sp. has a great ability to produce a wide of secondary metabolites which are considered a potential source of new anticancer compounds (Kusari et al., 2009; El-Sayed et al., 2021; Noman et al., 2021). A study conducted by Almanaa et al. (2021) reported that *A. fumigates* extract showed a cytotoxic effect against the HepG-2 cell line with IC_50_ value of 113 μg/mL. Also, the crude extract of *Aspergillus tubenginses* ASH4 showed anticancer effect against HCT-116, Hep-G2, and MCF-7 with IC_50_ values of 9.18, 10.41, and 5.89 μg/mL, respectively (Elkhouly et al., 2021a,b).

### UPLC-QTOF-MS/MS analysis

**Figure 10:**
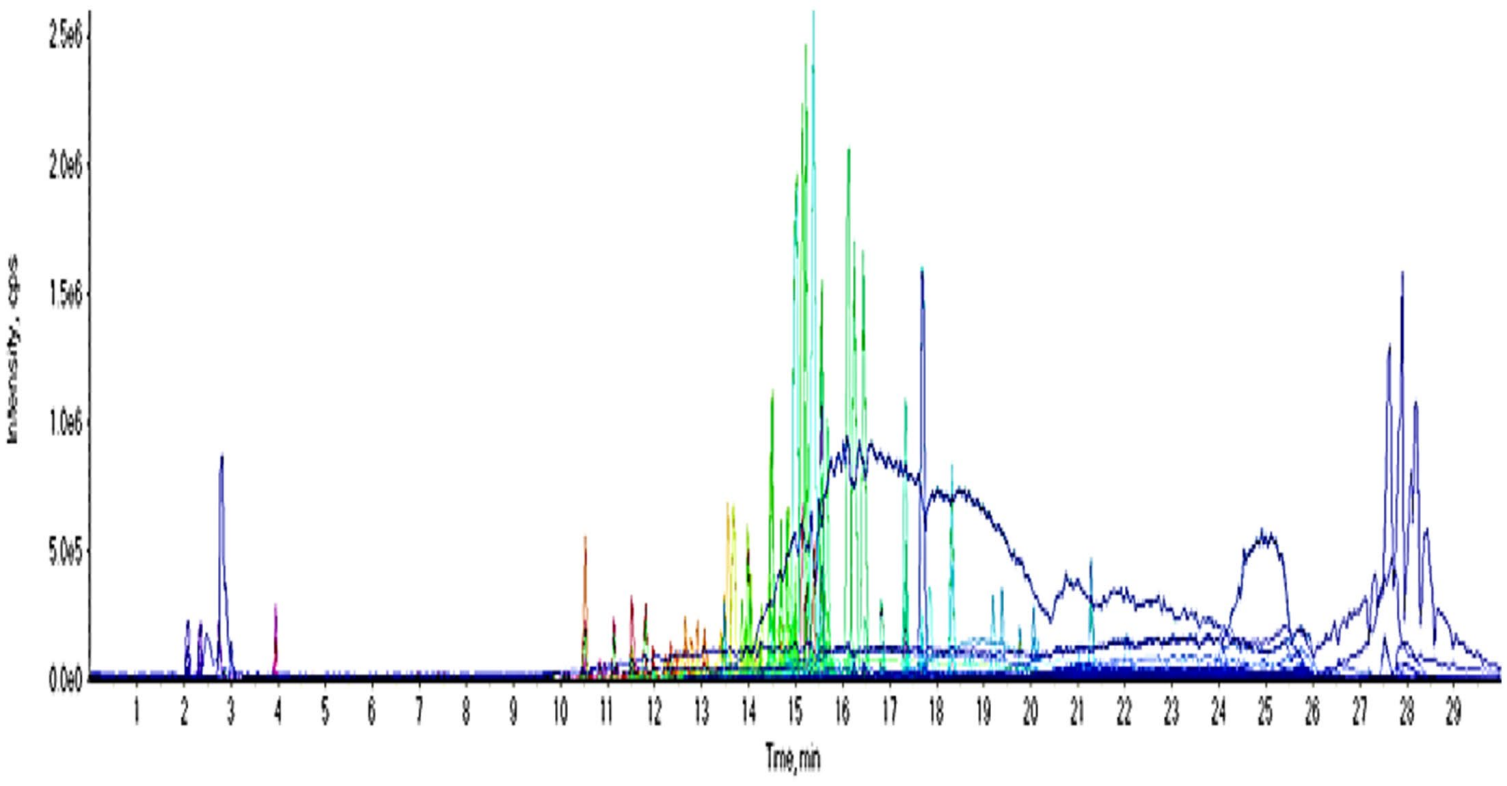
UPLC chromatogram of the crude extract in a negative ion mode.

UPLC-QTOF-MS/MS analysis of crude extract in a negative ion mode led to the identification of 33 compounds based on their retention times; fragmentation patters and via comparison with the available reported data. The identified compounds were categorized as carboxylic acids, phenolic acids, flavonoids, benzene derivatives, coumarins, anthraquinones, benzaldehydes, phenols and fatty acids (Figure 10; Table 1). The phenolic acids and flavonoids were the dominant compounds in the extract.

**Table 1:** Chemical constituents of the crude extract.

No.	Rt	[M-H]^−^	M.wt.	M.F.	Identified compound	Chemical class
1	2.04	149	150	C_4_H_6_O_6_	Tartaric acid	Carboxylic acids
2	2.07	117	118	C_5_H_10_O_3_	2-Hydroxy-2-methylbutyric acid	Carboxylic acids
3	2.38	391	392	C_25_H_28_O_4_	Glabrol	Flavonoids
4	2.42	255	256	C_15_H_12_O_4_	4,2′,5′-Trihydroxychalcone	Flavonoids
5	2.69	165	166	C_5_H_10_O_6_	Arabinonic acid	Carboxylic acids
6	2.94	191	192	C_10_H_8_O_4_	5,7-Dihydroxy-4-methylcoumarin	Hydroxycoumarins
7	3.07	413	414	C_23_H_26_O_7_	Garcinone C	Xanthones
9	3.94	137	138	C_7_H_6_O_3_	3-Hydroxybenzoic acid	Phenolic acids
10	10.15	343	344	C_19_H_20_O_6_	2’-Hydroxy-2,4,5,6′-tetramethoxychalcone	Flavonoids
11	10.25	165	166	C_9_H_10_O_3_	2’-Hydroxy-6′-methoxyacetophenone	Hydroxyacetophenones
12	10.49	225	226	C_14_H_10_O_3_	2-Benzoylbenzoic acid	Phenolic acids
13	10.64	167	168	C_8_H_8_O_4_	o-Vanillic acid	Phenolic acids
17	10.96	239	240	C_14_H_12_N_2_O_2_	β-Carboline-1-propionic acid	Alkaloids
14	11.51	419	420	C_25_H_24_O_6_	Pomiferin	Flavonoids
15	11.91	121	122	C_7_H_6_O_2_	3-Hydroxybenzaldehyde	Aldehydes
16	11.96	241	242	C_15_H_14_O_3_	Pinostilbene	Phenylpropanoids
17	12.18	625	626	C_27_H_30_O_17_	Quercetin 3,4′-di-*O*-glucoside	Flavonoids
18	12.23	183	184	C_8_H_8_O_5_	Methyl gallate	Phenolic esters
19	12.33	311	312	C_18_H_16_O_5_	3′,4′-Dimethoxy-3-hydroxy-6-methylflavone	Flavonoids
20	12.34	165	166	C_8_H_6_O_4_	Phthalic acid	Phenolic acids
21	13.38	493	494	C_24_H_30_O_11_	Harpagoside	Iridoid glycosides
22	13.44	207	208	C_10_H_8_O_5_	Fraxetin	Coumarins
23	13.48	269	270	C_15_H_10_O_5_	Emodin	Anthraquinones
24	14.05	207	208	C_11_H_12_O_4_	Ethyl caffeate	Phenolic esters
25	14.14	144	145	C_9_H_7_NO	Quinolin-8-ol	Alkaloids
26	14.23	327	328	C_18_H_16_O_6_	Kaempferol 3,7,4′-trimethyl ether	Flavonoids
27	14.28	253	254	C_15_H_10_O_4_	3,6-Dihydroxyflavone	Flavonoids
28	14.80	253	254	C_15_H_10_O_4_	Daidzein	Flavonoids
29	14.94	283	284	C_16_H_12_O_5_	Physcion	Anthraquinones
30	15.62	151	152	C_8_H_8_O_3_	Methyl 3-hydroxybenzoate	Phenolic esters
31	16.66	265	266	C_18_H_18_O_2_	Honokiol	Lignans
32	19.16	333	334	C_15_H_10_O_7_S	Daidzein 4′-sulfate	Flavonoids
33	21.33	595	596	C_27_H_32_O_15_	Eriocitrin	Flavonoids

#### Phenolic, organic and fatty acids and their derivatives

A peak demonstrated an [M-H]- *m/z* at 149 and daughter ions at *m/z* 105 [M-H-CO_2_]-, 87 [M-H-CO_2_-H_2_O]-, 71 and 59; it was assigned as tartaric acid (McGovern et al., 2009). A peak demonstrated an [M-H]- *m/z* at 117 and daughter ions at *m/z* 73 [M-H-CO_2_]-, 55 [M-H-CO_2_-H_2_O]-, and 61; it was assigned as 2-hydroxy-2-methylbutyric acid (John Wiley & Sons, Inc, 2015). A peak demonstrated an [M-H]- *m/z* at 317 and daughter ions at *m/z* 273 [M-H-CO_2_]-, 165, 113, and 103; it was assigned as 15-Oxo-5Z,8Z,11Z,13E-eicosatetraenoic acid (Sorgi et al., 2018). A peak demonstrated an [M-H]- *m/z* at 165 and a daughter ion at *m/z* 119 [M-H-HCOOH]-; it was assigned as arabinonic acid (Moreira et al., 2014). A peak demonstrated an [M-H]- *m/z* at 115 and a daughter ion at *m/z* 71 [M-H-CO_2_]-; it was assigned as 3-oxopentanoic acid (Kallem et al., 2021). A peak demonstrated an [M-H]- *m/z* at 137 and daughter ions at *m/z* 109 [M-H-CO]-, 108 [M-2H-CO]-, 93 [M-H-CO_2_]-, 92 [M-2H-CO_2_]-, 81, and 65; it was assigned as 3-hydroxybenzoic acid (Ammar et al., 2020; Ghareeb et al., 2023). Hydroxybenzoic acids as a subclass of phenolic acids possess a reliable role in managing neurodegenerative diseases aging, and cancer (e.g., breast cancer; Kalinowska et al., 2021). A peak demonstrated an [M-H]- *m/z* at 225 and daughter ions at *m/z* 207 [M-H-H_2_O]-, 179, 135, and 97; it was assigned as 2-benzoylbenzoic acid (Zengin et al., 2022). A peak demonstrated an [M-H]- *m/z* at 167 and daughter ions at *m/z* 152 [M-H-CH_3_]-, 123 [M-H-CO_2_]-, 108 [M-H-CH_3_-CO_2_]-, and 91 [M-H-COOH-OCH_3_]-; it was assigned as vanillic acid (Ghareeb et al., 2018a; Sayed et al., 2022). Vanillic acid, an oxidized form of vanillin, is a major active compound isolated from Angelica sinensis and green tea. Vanillin acid is a dietary phenol that can protect biofilms and inhibit lipid peroxidation and eliminates ROS hence act as anti-microbial, anti-inflammatory, anti-cancer factor (Gong et al., 2019). A peak demonstrated an [M-H]- *m/z* at 183 and daughter ions at *m/z* 169 [M-H-CH_3_]-, 168, 125 [M-H-CO_2_]-, 109, and 93; it was assigned as methyl gallate (Ghareeb et al., 2018b, 2019b). A peak demonstrated an [M-H]- *m/z* at 165 and daughter ions at *m/z* 121 [M-H-CO_2_]-, and 77 [M-H-2CO_2_]-; it was assigned as phthalic acid (Gillespie et al., 1989). A peak demonstrated an [M-H]- *m/z* at 207 and daughter ions at *m/z* 161 [M-H-C_2_H_5_OH]-; it was assigned as ethyl caffeate (Barth et al., 2019). A peak demonstrated an [M-H]- *m/z* at 151 and daughter ions at *m/z* 136 [M-H-CH_3_]-, and 92 [M-H-CH_3_-CO_2_]-; it was assigned as methyl 3-hydroxybenzoate (Hanley, 2006).

A peak demonstrated an [M-H]- *m/z* at 191 and daughter ions at *m/z* 176 [M-H-CH_3_]-, 147 [M-H- CO_2_]-, 160 [M-H-OH-CH_3_]-, 156 [M-H-2OH]-, and 105; it was assigned as 5,7-dihydroxy-4-methylcoumarin (Kerebba et al., 2022). A peak demonstrated an [M-H]- *m/z* at 207 and daughter ions at *m/z* 192 [M-H-CH_3_]-, 164 [M-H-CH_3_-CO]-, and 102; it was assigned as fraxetin (Wang et al., 2016). Fraxetin is a bioactive molecule present in various natural plants, considered as bioactive molecule that acts as anticancer, antioxidative, anti-inflammatory, antidiabetic and antimicrobial activities (Ha and Son, 2024).

#### Xanthones

A peak demonstrated an [M-H] *m/z* at 413 and daughter ions at *m/z* 323 [M-H-90]-, 285 [M-H-128]-, 257 [M-H-156]-, 229 [M-H-184]-; it was assigned as garcinone C (Khaw et al., 2020). Xanthones as secondary metabolites displayed various biological properties, including cytotoxic, antidiabetic, antioxidant, antimicrobial, antitumor, antihypertensive, and anti-inflammatory (Mohamed and Ibrahim, 2022).

#### Flavonoids

A peak demonstrated an [M-H]- *m/z* at 391 and daughter ions at *m/z* 203 [M-H-188]-, 187 [M-H-204]-, and 159 [M-H-188-44]-; it was assigned as glabrol (Simons et al., 2009). A peak demonstrated an [M-H]- *m/z* at 255 and daughter ions at *m/z* 135 [M-H-C_7_H_4_O_2_]-, and 119 [M-H-C_7_H_4_O_3_]-; it was assigned as 4,2′,5′-trihydroxychalcone (Shan et al., 2017). A peak demonstrated an [M-H]- *m/z* at 343 and daughter ions at *m/z* 315 [M-H-CO]-, 313 [M-H-2CH_3_]-, 328 [M-H-CH_3_]-, 300 [M-H-CH_3_-CO]-, and 297 [M-H-H_2_O-CO]-; it was assigned as 2′-hydroxy-2,4,5,6′-tetramethoxychalcone (Liu et al., 2016). A peak demonstrated an [M-H]- *m/z* at 419; it was assigned as pomiferin (Tian et al., 2006). A peak demonstrated an [M-H]- *m/z* at 625 and daughter ions at *m/z* 463 [M-H-Glc]^−^, and 301 [M-H-2Glc]-, 271, and 255; it was assigned as quercetin 3,4′-di-*O*-glucoside (Kumar et al., 2017). A peak demonstrated an [M-H]- *m/z* at 311 and daughter ions at *m/z* 293, and 209; it was assigned as 3′,4′-dimethoxy-3-hydroxy-6-methylflavone (Reed, 2009). A peak demonstrated an [M-H]^−^ *m/z* at 327 and daughter ions at *m/z* 314, 312, 297, 285, 284, 282, 270, and 112; it was assigned as kaempferol 3,7,4′-trimethyl ether (Dehkordi et al., 2020). A peak demonstrated an [M-H]- *m/z* at 253 and daughter ions at *m/z* 238, 223, 179, 151, and 123; it was assigned as 3,6-dihydroxyflavone (March and Brodbelt, 2008). A peak demonstrated an [M-H]- *m/z* at 253 and daughter ions at *m/z* 225 [M-H-CO]-, 224 [M-H-CHO]-, 209 [M-H-CO_2_]-, 197 [M-H-2CO]-, 185 [M-H-2CO + C]-, and 135 [M-H-C_8_H_6_O]-; it was assigned as daidzein (Zhao et al., 2018). A peak demonstrated an [M-H]- *m/z* at 333 and daughter ions at *m/z* 253 [M-H-SO_3_]-, 225 [M-H-SO_3_-CO]-, 224 [M-H-SO3-CHO]-, 209 [M-H-SO_3_-CO_2_]-, 197 [M-H-SO_3_-2CO]-, 185 [M-H-SO3-2CO + C]-, and 135 [M-H-SO_3_-C_8_H_6_O]; it was assigned as daidzein 4′-sulfate (Zhao et al., 2018). A peak demonstrated an [M-H]- *m/z* at 595 and daughter ions at *m/z* 433 [M-H-Glc]-, 287 [M-H-Glc-Rha]-; it was assigned as eriocitrin (Li et al., 2020).

#### Hydroxyacetophenones

A peak demonstrated an [M-H]- *m/z* at 165 and daughter ions at *m/z* 150 [M-H-CH_3_]-, 137 [M-H-CO]-, 122 [M-H-CO-CH_3_]-, and 107 [M-H-CO-2CH_3_]-; it was assigned as 2′-hydroxy-6′-methoxyacetophenone (Martin, 2013).

#### Alkaloids

A peak demonstrated an [M-H]- *m/z* at 239 and daughter ions at *m/z* 221 [M-H-H_2_O]-, 193 [M-H-CH_2_O_2_]-, 179 [M-H-C_2_H_4_O_2_]-, 165 [M-H-C_3_H_6_O_2_]-, and 138 [M-H-C_4_H_7_NO_2_]-; it was assigned as β-carboline-1-propionic acid (Chua et al., 2011). A peak demonstrated an [M-H]- *m/z* at 144 and daughter ions at *m/z* 114, and; it was assigned as quinolin-8-ol (Clugston, 1996).

#### Hydroxybenzaldehydes

A peak demonstrated an [M-H]- *m/z* at 121 and daughter ions at *m/z* 93 [M-H-CO]- and 92 [M-H-CHO]-; it was assigned as 3-hydroxybenzaldehyde (Castillo et al., 2023).

#### Phenylpropanoids

A peak demonstrated an [M-H]- *m/z* at 241 and daughter ions at *m/z* 225, 197, 181, and 169; it was assigned as pinostilbene (Chen et al., 2016).

#### Iridoids

A peak demonstrated an [M-H]- *m/z* at 493 and daughter ions at *m/z* 363 [M-H-cinnamoyl]-, 345 [M-H-cinnamoyl-H_2_O]-, 201 [M-H-cinnamoyl-Glc]-, 183 [M-H-cinnamoyl-H_2_O-Glc]-, and 147 [M-H-C_15_H_22_O_9_]-; it was assigned as harpagoside (Xiong et al., 2010).

#### Anthraquinones

A peak demonstrated an [M-H]- *m/z* at 269 and daughter ions at *m/z* 251 [M-H-H_2_O]-, 241 [M-H-CO]-, 227 [M-H-CO-CH_2_]-, 225, 223 [M-H-CO-H_2_O]-, 195 [M-H-CO-H_2_O-CO]-; it was assigned as emodin (Fu et al., 2015). A peak demonstrated an [M-H]- *m/z* at 283 and daughter ions at *m/z* 268 [M-H-CH_3_]-, 240 [M-H-CH_3_-CO]-, 212, and 184; it was assigned as physcion (Fu et al., 2015).

#### Lignans

A peak demonstrated an [M-H]- *m/z* at 265 and a daughter ion at *m/z* 224 [M-CH_2_CH=CH_2_-H]-; it was assigned as honokiol (Wu et al., 2006).

### *In silico* studies

#### Virtual screening-based target identification

To find out how crude extract exerts its antibacterial and anticancer activities, all the modeled structures of the UPLC–MS-annotated compounds in these samples were subjected to pharmacophore-based virtual using the PharmMapper platform (Wang et al., 2017).

PharmMapper can screen and recommend the most probable protein targets of a query molecule based on its pharmacophore model by mapping its fundamental pharmacophore characteristics (i.e., the spatial arrangement of structural features).

Accordingly, the compounds that are compatible with these pharmacophore maps have a greater potential for binding to the same protein targets. Therefore, the annotated compounds (Table 2) were run through PharmMapper to find their potential protein targets. The obtained results were arranged according to their degree of conformity to the pre-determined parameters (the Fit score). Only bacterial-relevant and cancer-relevant targets were chosen.

**Table 2:** Docking scores and Δ*G*_Bind_ (in kcal/mol) of glabrol and pomiferin inside *Escherichia coli* GyrB’s active sites.

Compounds	Docking score	MM-PBSA(ΔG_Bind_)	H-Bonds	Hydrophobic interactions
Glabrol	−11.48	−9.89	ASP-73, ARG-76, THR-165	PRO-79, ILE-94, ILE-98, ALA-100, VAL-120
Pomiferin	−10.13	−9.08	ARG-76, GLY-77	PRO-79, ILE-94, ILE-98, VAL-120
Co-crystalized inhibitor	−11.54	−7.79	ASP-73	PRO-79, ILE-94, ILE-98, VAL-120

**Figure 11:**
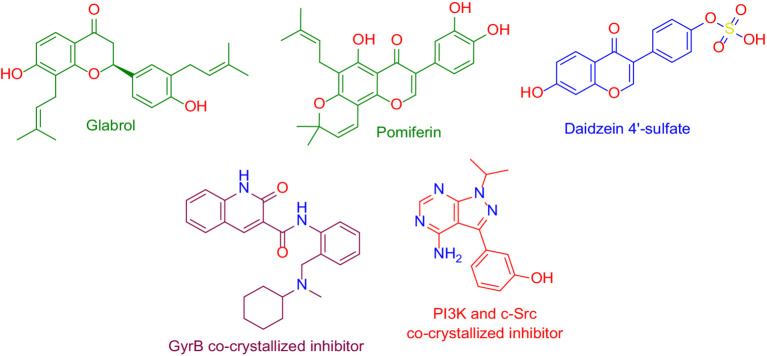
Structures that were found to be probably able to bind to the active sites of *Escherichia coli*’s GyrB (i.e., glabrol and pomiferin; green-colored structures), and PI3K (i.e., daidzein 4′-sulfate, blue-colored structure), according to the preliminary PharmMapper-based virtual screening alongside the GyrB and PI3K-γ co-crystalized inhibitors (purple-colored and orange-colored structures).

As a result, DNA gyrase subunit-B (GyrB) of *E. coli* (PDB ID: 6KZV) was found to be the top-scoring bacterial-relevant hit for glabrol and pomiferin (Figure 11). Hence, these metabolites can be considered tentatively as the key antibacterial metabolites in the tested extract.

On the other hand, Daidzein 4′-sulfate (Figure 11) was predicted to bind with the active site of human phosphoinositide 3-kinase gamma (PI3K-γ; PDB ID: 2V4L) with a Fit score of 8.28.

It is well-known that GyrB is an essential protein that provides the necessary energy for the GyrA subunit, which in turn unfolds the bacterial DNA, making it available for the DNA replicating enzyme, and thus blocking these enzymes (i.e., GyrA or GyrB) will stop the bacterial DNA replication and eventually bacterial death (Wigley et al., 1991; Ushiyama et al., 2020).

Regarding the cancer-relevant protein, PI3K-γ has been reported in many previous reports to be over-expressed in different types of human tumors, particularly breast cancers (Apsel et al., 2008; Kwak et al., 2019; Miricescu et al., 2020).

#### Molecular docking and dynamics simulation analysis

To investigate the binding modes of each aforementioned compound (Figure 11) with GyrB and PI3K, their modeled structures were prepared and re-docked into the active sites of each protein. Thereafter, the resulting binding poses were subjected to 50 ns-long MD simulation runs to test the binding affinity and stability of each structure inside the active sites of the suggested protein targets. First, the re-docking of glabrol and pomiferin structures into the GyrB’s active site achieved binding modes and docking scores comparable to those of the co-crystalized inhibitor (Figure 11; Table 2).

**Figure 12:**
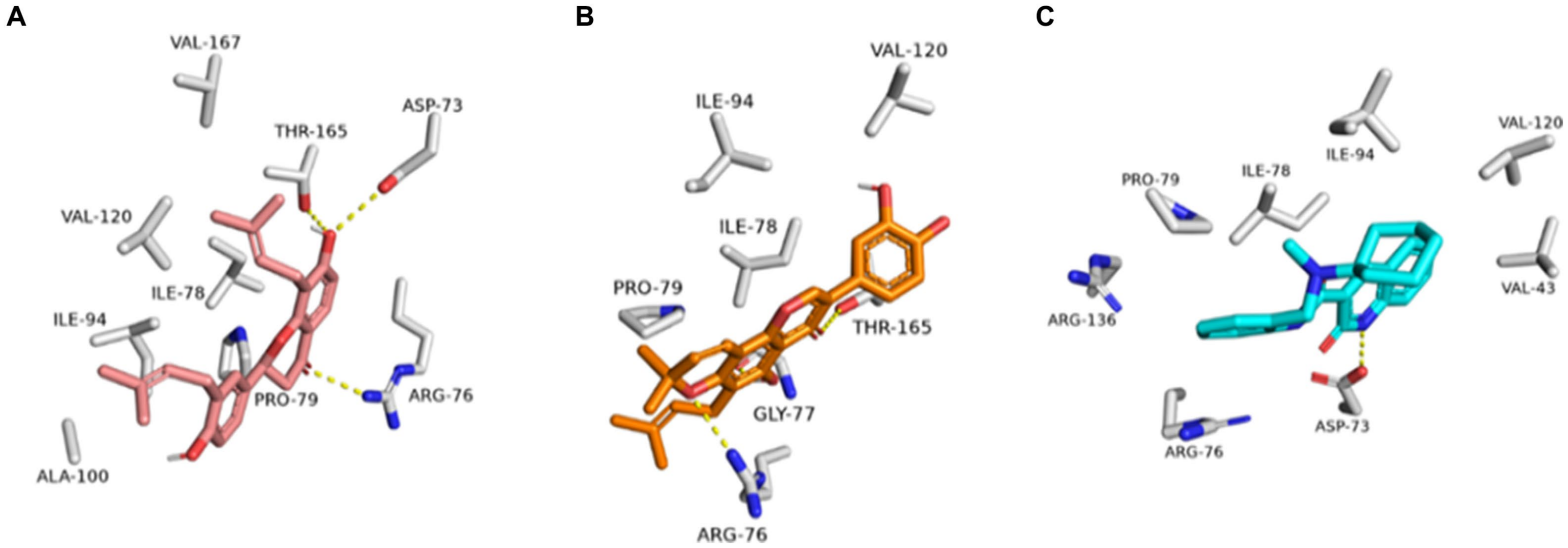
Binding modes of glabrol (brick red-colored structure) **(A)**, and pomiferin (orange-colored structure) **(B)** along with the co-crystalized inhibitor (cyan-colored structure) **(C)** inside *E. coli* GyrB’s active site.

**Figure 13:**
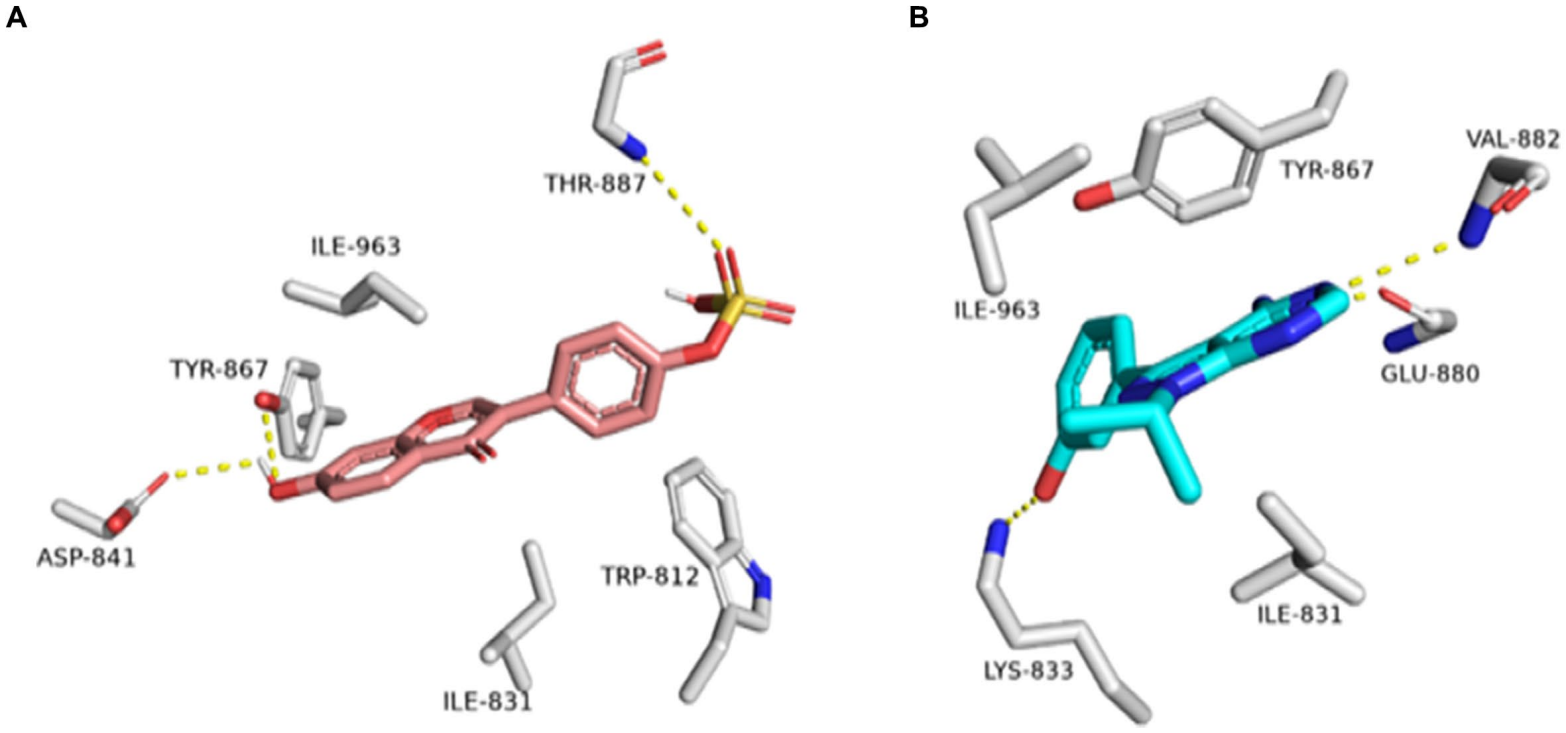
Binding modes of daidzein 4′-sulfate along with the co-crystalized inhibitor (**A**: brick red-colored structures and **B**: cyan-colored structure, respectively) inside the active site of PI3K.

**Figure 14:**
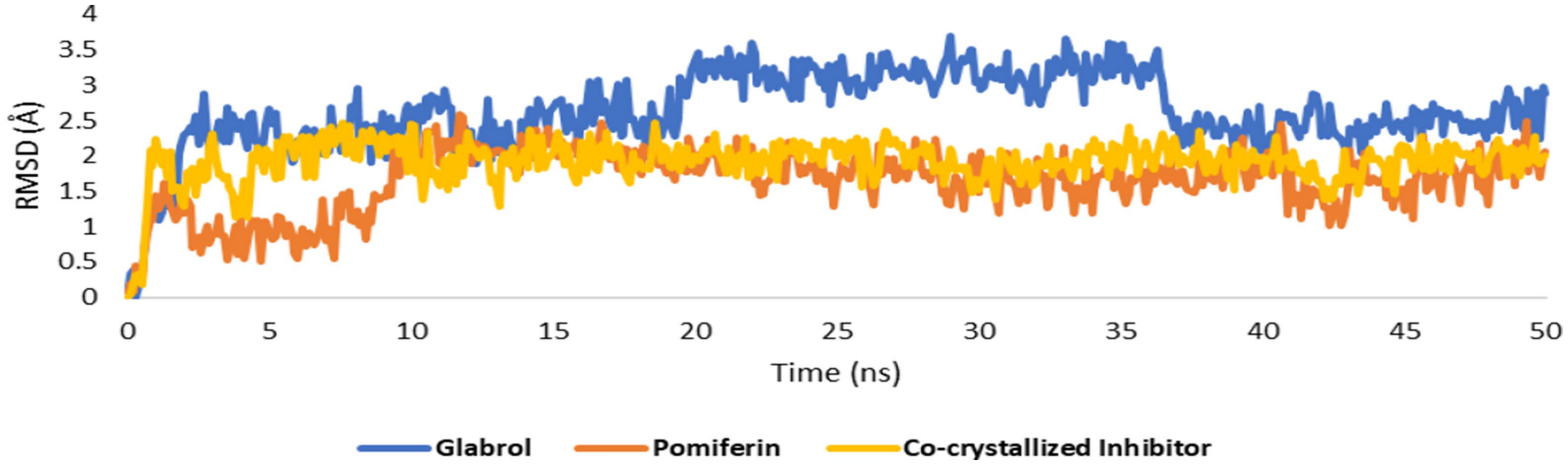
RMSDs of glabrol and pomiferin structures along with that of the co-crystallized inhibitor inside the GyrB’s active site throughout 50 ns-long MD simulation.

As shown in Figures 10A–C, the structures of glabrol and pomiferin were able to achieve binding modes convergent to that of the co-crystalized inhibitor forming comparable hydrophilic and hydrophobic interactions (Table 2). H-bonds with ASP-73 and ARG-76 were the common hydrophilic interactions among the three structures alongside the co-crystalized inhibitor (Figure 12). Second, re-docking of daidzein 4′-sulfate structure into the active sites of PI3K resulted in binding mode and docking score comparable to those of the co-crystalized inhibitor (Figure 13; Table 3). Subsequent experiments using molecular dynamic (MD) simulation (lasting 50 ns) showed that glabrol and pomiferin structures were able to achieve stable binding modes inside the GyrB’s active site, with relative mean square deviations (RMSDs) of 2.523 Å and 1.765 Å, respectively (Figure 14). Hence, the calculated binding free energies (Δ*G*_Binding_) these structures in comparison with that of the co-crystallized inhibitor were convergent (Δ*G*_Binding_ = −7.57, −8.54, and −8.76 kcal/mol, respectively).

**Table 3:** Docking scores and Δ*G*_Bind_ (in kcal/mol) of daidzein 4′-sulfate into the active site of PI3K.

Compounds	Docking score	MM-PBSA(ΔG_Bind_)	H-Bonds	Hydrophobic interactions
Daidzein 4′-sulfate with PI3K	−8.44	−7.59	ASP-841, TYR-867, THR-887	TRP-812, ILE-831, ILE-963
Co-crystalized inhibitor with PI3K	−11.19	−9.19	LYS-833, GLU-880, VAL-882	TRP-812, ILE-831, TYR-867, ILE-963

**Figure 15:**
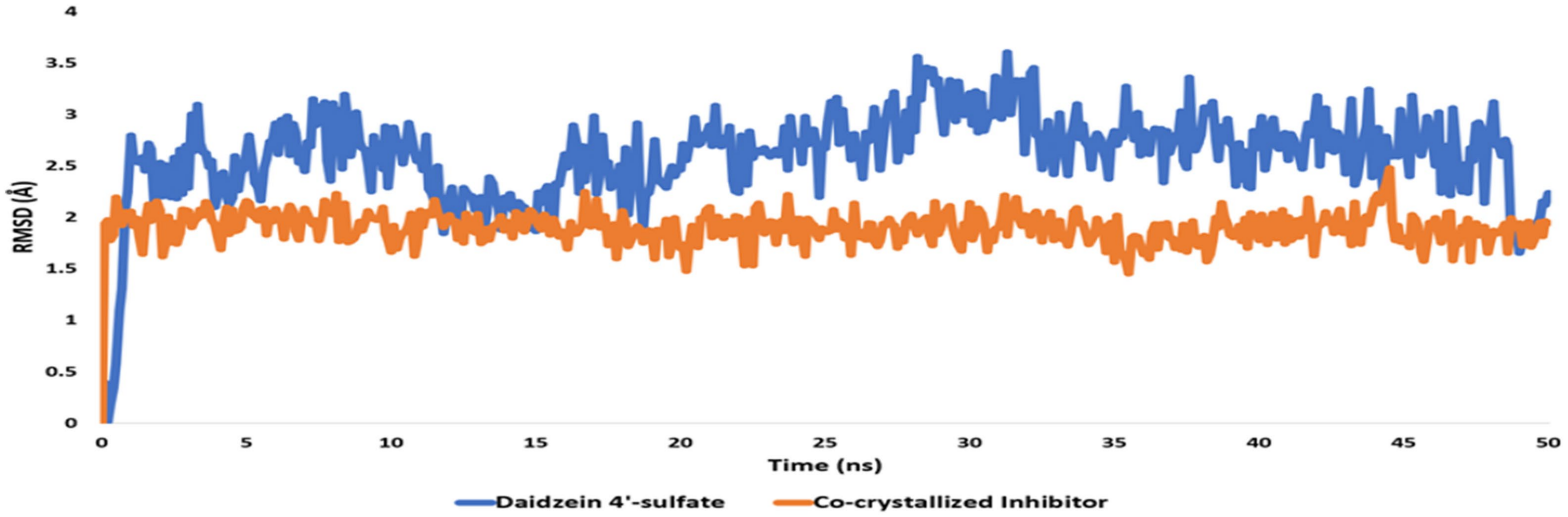
RMSDs of daidzein 4′-sulfate structure alongside that of the co-crystallized inhibitor inside the active site of PI3K throughout 50 ns-long MD.

Regarding the binding behavior of daidzein 4′-sulfate structure inside the active sites of PI3K throughout 50 ns-long MD simulations in comparison with the co-crystallized inhibitor, it also achieved stable binding concerning the co-crystallized inhibitor with an average RMSD of 2.552 Å. Additionally, their calculated Δ*G*_Binding_ values were convergent to that of the co-crystalized inhibitor (Δ*G*_Binding_ = −8.96 and −8.87 kcal/mol for daidzein 4′-sulfate and the co-crystallized inhibitor, respectively; Figure 15).

Moreover, modeling and MD simulation results suggest that glabrol and pomiferin in the tested extract are the antibacterial-relevant metabolites, while daidzein 4′-sulfate is the anticancer-relevant metabolite. In previous reports, glabrol and pomiferin have been shown to exert interesting antibacterial efficacies vs. both Gram-positive and Gram-negative pathogenic bacteria including antibiotics-resistant ones (Gerhäuser, 2005; Stompor and Żarowska, 2016; Wu et al., 2019; Mohamed et al., 2022).

## Discussion

Antimicrobial resistance (AMR) has gained global attention in recent years due to the terrifying prospect of rising death rates. Nanomaterials are being studied because of their potential in a variety of technical and biological applications. Zinc nanoparticles (ZnONPs) have attracted reasonable interest as potential candidates for usage in a variety of sectors including medicine, industry, and agriculture. The fungal strain SA17 was isolated and identified based on its morphological characteristics and genetically by sequencing of the 18S rRNA gene as Aspergillus sp. SA17. The fungal crude extract was obtained and used in the green biosynthesis of zinc oxide nanoparticles (ZnONPs). Numerous investigations revealed that after heating the zinc ions to 100°C, the enzymes and biomolecules in the fungal filtrate function as reducing agents to transform the zinc ions into zinc oxide nanoparticles in solution. By adjusting the temperature, incubation period, and precursor solution concentration, one may control the size and form of the nanoparticles. The UV–Vis absorption spectrum provides critical insights into the electronic changes occurring in the produced nanoparticles. The detected absorption bands correspond to specific electronic transitions and can provide information about the nanoparticles’ size, structure, and optical properties (Hameed et al., 2023). The results show that the reduced size of the nanoparticles generates a quantum confinement effect, as indicated by the appearance of an absorption band in the UV region (Qin and Zeng, 2017). In this case, the presence of larger particles or agglomerates is indicated by the absorption band at 340 nm, while the presence of small particles is indicated by the absorption peak at 280 nm (Mitjans et al., 2023). The broad absorption profile that is frequently observed in nanomaterials is caused by the variety of particle sizes in the produced sample. Additionally, the zeta potential value gives important information regarding the surface charge characteristics of ZnONPs, implying their potential for stable dispersion in neutral aqueous environments. Our data was very similar to those reported by Abdelbaky et al. (2022) and Abdelgawad et al. (2023) and more stable than ZnO nanoparticles prepared by Iqbal et al. (2021). The FTIR analysis was also utilized to study the functional groups in charge of ZnONPs production and stability including CH, C=O, C=C, C-O, C-N, and C-C-. while X-ray diffraction (XRD) was used to confirm the crystalline nature of ZnONPs. Moreover, TEM and SEM were done to investigate the nature of SA-17 ZnONPs particle size distribution and its crystallinity.

Biological evaluation of the crude extract and biosynthesized ZnONPs as antimicrobial was investigated toward several pathogens and results showed a pronounced antimicrobial effect toward *E. coli*, *P. aeruginosa*, *S. aureus*, *B. subtilis*, *C. albicans*, and *A. flavus*, respectively. The obtained results were compared with standard antibiotics, including Ciprofloxacin and Colitrimazole. *Aspergillus* species are known for producing unique and diverse secondary metabolites with potent biological activities (Zhang et al., 2018; Abdel-Razek et al., 2020). Several studies have demonstrated the vital role of some fungal metabolites with antimicrobial activity such as phenolic acids, flavonoids, coumarins, and anthraquinones (Nagia et al., 2012; Beekman and Barrow, 2014; Li et al., 2023). Rodrigues et al. (2022) reported that the ethyl acetate crude extract of *Aspergillus* sp. has shown antimicrobial activity against *E. coli* CBAM, *S. aureus* CBAM, *S. aureus* ATCC, and *S. aureus* MRSA with inhibition zone diameter values of 14, 10, 7, and 9 mm, respectively. Moreover, the ethyl acetate extract from *Aspergillus unguis* SPMD-EGY exhibited a marked antimicrobial effect against *S. aureus*, *P. aeruginosa*, and *C. albicans* (Hamed et al., 2018). In another study, the ethyl acetate extract of *Aspergillus fumigatus* 3 T-EGY showed an antimicrobial effect against *S. aureus*, *P. aeruginosa*, *C. albicans*, and *A. niger* with inhibition zone diameter values of 10, 15, 15, and 9 mm, respectively (Abdel-Aziz et al., 2018). Additionally, the dichloromethane extract of *Aspergillus tubenginses* ASH4 exhibited an antimicrobial effect on *P. aeruginosa*, *S. aureus*, *E. coli*, *B. subtilis*, and *C. albicans* with inhibition zone diameter values of 14, 15, 16, 13 and 15 mm, respectively (Elkhouly et al., 2021a). Furthermore, Elkhouly et al. (2021b) stated that the dichloromethane extract of *Aspergillus terreus* AH1 exhibited antimicrobial efficacy against *C. albicans*, *S. aureus*, *B. subtilis*, *P. aeruginosa*, and *E. coli* with inhibition zone diameter values of 17, 16, 14, 15, and 16 mm, respectively. Consequently, the results of the extract under study are to some extent consistent with previous studies. On the other side, several previous studies reported on the antimicrobial efficacy of the biosynthesized ZnONPs against a broad array of microbial strains. For instance, the *Camellia japonica* leaf extract zinc oxide nanoparticles showed antibacterial effect against *S. pneumoniae*, *B. subtilis*, *E. coli*, and *S. typhimurium* with inhibition zone values of 21, 13.5, 19, and 14 mm, respectively (Maruthupandy et al., 2018). Also, these ZnONPs showed antifungal effect against *A. flavus*, *A. fumigatus*, *A. niger*, and *C. albicans* with inhibition zone values of 9.6, 10.5, 13, and 19.1 mm, respectively (Maruthupandy et al., 2018). We can conclude that the tested ZnONPs in our current study showed low antimicrobial efficacy when compared with the findings of Muthuchamy et al. (2020). Also, our results were to some extent with the previous findings (Maruthupandy et al., 2018; Rajivgandhi et al., 2018). On the other hand, The crude extract demonstrated anticancer activity with IC_50_ values of (84.55 μM), (31.13 μM), (39.06 mm), and (17.65 μM), against WI38, HCT116, HePG2, and MCF7, respectively. On the other hand, the biosynthesized ZnONPs displayed anticancer activity with IC_50_ values of (59.74 μM), (43.21 μM), (57.03 mm), and (35.66 μM), against WI38, HCT116, HePG2, and MCF7, respectively. The results were compared with Doxorubicin (Figure 9). *Aspergillus* sp. has a great ability to produce a wide of secondary metabolites which are considered a potential source of new anticancer compounds (Kusari et al., 2009; El-Sayed et al., 2021; Noman et al., 2021). A study conducted by Almanaa et al. (2021) reported that *A. fumigates* extract showed a cytotoxic effect against the HepG-2 cell line with IC_50_ value of 113 μg/mL. Also, the crude extract of *Aspergillus tubenginses* ASH4 showed anticancer effect against HCT-116, Hep-G2, and MCF-7 with IC_50_ values of 9.18, 10.41, and 5.89 μg/mL, respectively (Elkhouly et al., 2021a,b). Additionally, the crude extract and biosynthesized ZnONPs demonstrated anticancer activity against WI38, HCT116, HePG2, and MCF7. *Aspergillus* sp. has a great ability to produce a wide range of secondary metabolites which are considered a potential source of new anticancer compounds (Kusari et al., 2009; El-Sayed et al., 2021; Noman et al., 2021). A study conducted by Almanaa et al. (2021) reported that *A. fumigates* extract showed a cytotoxic effect against the HepG-2 cell line with IC_50_ value of 113 μg/mL. Also, the crude extract of *Aspergillus tubenginses* ASH4 showed anticancer effect against HCT-116, Hep-G2, and MCF-7 with IC_50_ values of 9.18, 10.41, and 5.89 μg/mL, respectively (Elkhouly et al., 2021a,b). Chemical profiling of the extract using UPLC-QTOF-MS/MS also revealed 33 components, including flavonoids, phenolic acids, coumarins, organic acids, anthraquinones, and lignans. Based on previous reports, the different extracts of *Aspergillus* sp., were screened for their chemical profiles using various chromatographic and spectroscopic tools especially the hyphenated systems like UPLC-QTOF-MS/MS technique (Abdel-Aziz et al., 2018; Abdelgawad et al., 2022). Many compounds have been identified and/ or isolated belonging to several chemical classes such as anthraquinones, phenolic acids, flavonoids, coumarins, alkaloids, lactones, and terpenes (Hussein et al., 2022; Tang et al., 2022; Magot et al., 2023), indicating their unique chemical composition, which is reflected in the biological activities of these extracts. An *in silico* study suggested that pomiferin and glabrol could have antibacterial properties, and that daidzein 4′-sulfate could be a promising anti-cancer metabolite.

## Conclusion

The current study illustrates the manufacture and analysis of zinc nanoparticles utilizing *Aspergillus* sp. SA17 extract. These nanoparticles demonstrate potential antibacterial and anticancer cell properties, which could have major biomedical uses. Furthermore, the produced zinc nanoparticles’ proven antibacterial and anticancer capabilities open the door for further research into their precise mechanisms of action and potential benefits when combined with current treatment approaches. The chemical examination of the extract indicated chemicals that could be useful in treating bacterial infections and cancer. These discoveries open up new avenues for future research and growth in these fields. Overall, our findings contribute to the growing body of knowledge about tailored nanoparticles and their wide uses in a variety of sectors.

## Data availability statement

The original contributions presented in the study are included in the article/Supplementary material, further inquiries can be directed to the corresponding authors.

## Author contributions

SA: Methodology, Investigation, Writing – original draft, Writing – review & editing. SE: Conceptualization, Writing – original draft, Writing – review & editing, Supervision, Project administration. EM: Conceptualization, Validation, Writing – review & editing. ME: Methodology, Investigation & Writing – original draft. HS: Visualization, Writing – review & editing. AHamd: Visualization, Writing – review & editing. MG: Conceptualization, Methodology, Validation, Formal analysis, Investigation, Writing – original draft, Writing – review & editing, Supervision, Project administration. AHame: Conceptualization, Methodology, Validation, Formal analysis, Investigation, Writing – original draft, Writing – review & editing, Supervision, Project administration.
