# Supplementary material for: Bio-fabricated zinc oxide nanoparticles mediated by endophytic fungus Aspergillus sp. SA17 with antimicrobial and anticancer activities: in vitro supported by in silico studies
Source: Front Microbiol. 2024 May 13;15:1366614. doi: 10.3389/fmicb.2024.1366614 (PMC11128569; doi:10.3389/fmicb.2024.1366614)
Supplement: Supplementary file 1 [file Table_1.DOCX]

Supplementary Material

**Bio-fabricated Zinc Oxide Nanoparticles Mediated by Endophytic Fungus Aspergillus Sp. SA17 with Antimicrobial and Anticancer Activities: In Vitro Supported by In Silico Studies**

**Sally El Said Abo Halawa Abdelrahman ^1^, Seham El Hawary ^1^, Engy Mohsen ^1,*^, Mohamed A. El Raey ^2^, Heba Mohammed Refat M. Selim ^3,4^,** **Ahmed M. E. Hamdan ^5^, Mosad A. Ghareeb ^6^ , Ahmed A. Hamed^7^**

^1^Pharmacognosy Department, Faculty of Pharmacy, Cairo University, Egypt

^2^Department of Phytochemistry and Plant Systematics, Pharmaceutical Division, National Research Centre, 33 El Bohouth Street, P.O. Box 12622, Dokki, Cairo 12622, Egypt

^3^Department of Pharmaceutical Sciences, Faculty of Pharmacy, Almaarefa University, Diriyah, 13713, Riyadh, Saudi Arabia

^4^Microbiology and Immunology Department, Faculty of Pharmacy (Girls); Al-Azhar University, Cairo 35527, Egyp

^5^Department of Pharmacy Practice, Faculty of Pharmacy, University of Tabuk, Tabuk 71491, Saudi Arabia

^6^Medicinal Chemistry Department, Theodor Bilharz Research Institute, Kornish El-Nile, Warrak El-Hadar, Imbaba, Giza 12411, Egypt

^7^Microbial Chemistry Department, National Research Centre, 33 El-Buhouth Street, Dokki, Giza 12622, Egypt

***Correspondence:** ahmedshalbio@gmail.com (A.A.H.); engy.mohsen@pharma.cu.edu.eg (E.M.); Tel.: +20-15-000-002 (A.A.H.)

**Keywords: *Aspergillus* sp. SA17, ZnONPs, Antimicrobial, Anticancer, UPLC-QTOF-MS/MS, Docking, DNA gyrase, Phosphoinositide 3-kinase gamma**

**Abstract**

In recent years, the world's attention has been drawn to antimicrobial resistance (AMR) because to the frightening prospect of growing death rates. Nanomaterials are being investigated due to their potential in a wide range of technical and biological applications. The purpose of this study was to biosynthesis zinc oxide nanoparticles (ZnONPs) using Aspergillus sp. SA17 fungal extract, followed by characterization of the produced nanoparticles (NP) using electron microscopy (TEM and SEM), UV-analysis, X-ray diffraction (XRD), and Fourier-transform infrared spectroscopy (FT-IR). The HR-TEM revealed spherical nanoparticles with an average size of 7.2 nm, and XRD validated the crystalline nature and crystal structure features of the generated ZnONPs, while the zeta potential was 18.16 mV, indicating that the particles' surfaces are positively charged. The FT-IR was also used to identify the biomolecules involved in the synthesis of ZnONPs. The antibacterial and anticancer properties of both the crude fungal extract and its nano-form against several microbial strains and cancer cell lines were also investigated. Inhibition zone diameters against pathogenic bacteria ranged from 3 to 13 mm, while IC_50_ values against cancer cell lines ranged from 17.65 to 84.55 M. Additionally, 33 compounds, including flavonoids, phenolic acids, coumarins, organic acids, anthraquinones, and lignans, were discovered through chemical profiling of the extract using UPLC-QTOF-MS/MS. Some molecules, such pomiferin and glabrol, may be useful for antibacterial purposes, according to in silico study, while daidzein 4'-sulfate showed promise as an anti-cancer metabolite.

# Material and method of In silico study

***Virtual target identification***

The putative target characterization was achieved *via* Pharmacophore-based Virtual screening using PharmMapper (Wang et al., 2017). This platform assigns a score to each molecule in the PDB that best fits a pharmacophore model that has been extracted and stored as a library of ligand dataset in mol2 format. After that, when a new molecule is submitted, its fit score is calculated for each pharmacophore, and then each fit score for that pharmacophore is compared to the fit score matrix to determine where it falls on the scale of all the pharmacophore scores. In comparison to chance pharmacophore matching, the pure fit score that results from this procedure carries considerably more weight and assurance. The query structure was submitted to the platform in the PDB format, and the retrieved results were exported as Excel sheet arranging the resulted protein targets according their fit scores.

*Docking study*

The crystal structures of *E. coli* GyrB (PDB ID: 6kzv), and both the human PI3K-γ and c-Src (PDB ID: 2v4l and 3en7, respectively) were used for the docking study using AutoDock Vina (Huey et al., 2012). The co-crystallized ligand in each structure was used to determine the binding site and the docking grid-box in each protein structure, respectively. The co-ordinates of the grid-box were set to be: x= -7.86, y= 16.12, z= 2.49; and x= 45.07, y= 13.12, z= 31.49; and x= -5.09, y = 6.34, z = -6.66, respectively. The ligand to binding site shape matching root means square (RMSD) threshold was set to 2.0 Å. The interaction energies were determined using the Charmm force field (v.1.02) with 10.0 Å as a non-bonded cutoff distance and distance-dependent dielectric. Then, 5.0 Å was set as an energy grid extending from the binding site (Huey et al., 2012). The tested compound retinol was energy minimized inside the selected binding pocket. The editing and visualization of the generated binding poses were performed using Pymol software (Yuan et al., 2017).

*Molecular dynamics simulation*

NAMD 3.0.0. software was used for performing MDS (Yuan et al., 2017; Ribeiro et al., 2018)*.* This software applies the Charmm-36 force field. Protein systems were built using the QwikMD toolkit of the VMD software (Ribeiro et al., 2018;Humphrey et al., 2015)*.*, where the protein structures were checked for any missing hydrogens, the protonation states of the amino acid residues were set (pH = 7.4), and the co-crystalized water molecules were removed. Thereafter, the whole structures were embedded in an orthorhombic box of TIP3P water together with 0.15 M Na^+^ and Cl^-^ ions in 20 Å solvent buffer. Afterward, the prepared systems were energy minimized and equilibrated for 5 ns. The parameters and topologies of the ligands were calculated by using the VMD plugin Force Field Toolkit (ffTK). Afterward, the generated parameters and topology files were loaded to VMD to readily read the protein–ligand complexes without errors and then conduct the simulation steps.

*Binding free energy calculations*

Molecular Mechanics Poisson-Boltzmann Surface Area (MM-PBSA) embedded in the MMPBSA.py module of AMBER18 was utilized to calculate the binding free energy of the docked complex (Humphrey et al., 2015)*.* 100 frames were processed from the trajectories in total, and the system's net energy was estimated using the following equation:

ΔG_Binding_ = ΔG_Complex_ – ΔG_Receptor_ – ΔG_Inhibitor_

Each of the terms requires the calculation of multiple energy components, including van der Waals energy, electrostatic energy, internal energy from molecular mechanics, and polar contribution to solvation energy.

**References**

Wang, X., Shen, Y., Wang, S., Li, S., Zhang, W., Liu, X., Lai, L., Pei, J., Li, H. (2017). PharmMapper 2017 Update: A Web Server for Potential Drug Target Identification with a Comprehensive Target Pharmacophore Database. *Nucleic Acids Res.* 45, W356-W360. [DOI: 10.1093/nar/gkx374](https://doi.org/10.1093/nar/gkx374).

Huey, R., Morris, G. M., Forli, S. (2012). Using AutoDock 4 and AutoDock Vina with AutoDockTools: A Tutorial. *The Scripps Research Institute Molecular Graphics Laboratory.* 10550(92037), 1000.

Yuan, S., Chan, H. S., Hu, Z. (2017). Using PyMOL as a Platform for Computational Drug Design. *Wiley Interdiscip. Rev. Comput. Mol. Sci.* 7(2), e1298. [DOI: 10.1002/wcms.1298](https://doi.org/10.1002/wcms.1298).

Ribeiro, J. V., Bernardi, R. C., Rudack, T., Schulten, K., Tajkhorshid, E. (2018). QwikMD-Gateway for Easy Simulation with VMD and NAMD. *Biophys. J.* 114(3), 673a-674a. [DOI: 10.1016/j.bpj.2017.11.3632](https://doi.org/10.1016/j.bpj.2017.11.3632).

Humphrey, W., Dalke, A., Schulten, K. (1996). VMD: Visual Molecular Dynamics. *Journal of Molecular Graphics.* 14(1), 33-38.
